# Exploring transcriptional regulators Ref-1 and STAT3 as therapeutic targets in malignant peripheral nerve sheath tumours

**DOI:** 10.1038/s41416-021-01270-8

**Published:** 2021-03-03

**Authors:** Silpa Gampala, Fenil Shah, Chi Zhang, Steven D. Rhodes, Olivia Babb, Michelle Grimard, Randall S. Wireman, Ellie Rad, Brian Calver, Ren-Yuan Bai, Verena Staedtke, Emily L. Hulsey, M. Reza Saadatzadeh, Karen E. Pollok, Yan Tong, Abbi E. Smith, D. Wade Clapp, Andrew R. Tee, Mark R. Kelley, Melissa L. Fishel

**Affiliations:** 1 Department of Pediatrics and Herman B Wells Center for Pediatric Research, Indiana University, School of Medicine, Indianapolis, IN USA; 2 Department of Medical and Molecular Genetics, Indiana University, School of Medicine, Indianapolis, IN USA; 3 Department of Electrical and Computer Engineering, Purdue University, West Lafayette, IN USA; 4 Division of Cancer and Genetics, Cardiff University, Cardiff, Wales UK; 5 Neurosurgery and Neurology, Johns Hopkins School of Medicine, Baltimore, MD USA; 6 Department of Pathology and Laboratory Medicine, Indiana University, School of Medicine, Indianapolis, IN USA; 7 Department of Pharmacology and Toxicology, Indiana University, School of Medicine, Indianapolis, IN USA; 8 Department of Biostatistics and Data Management, Indiana University, School of Medicine, Indianapolis, IN USA

**Keywords:** Paediatric cancer, Sarcoma

## Abstract

**Background:**

MPNST is a rare soft-tissue sarcoma that can arise from patients with NF1. Existing chemotherapeutic and targeted agents have been unsuccessful in MPNST treatment, and recent findings implicate STAT3 and HIF1-α in driving MPNST. The DNA-binding and transcriptional activity of both STAT3 and HIF1-α is regulated by Redox factor-1 (Ref-1) redox function. A first-generation Ref-1 inhibitor, APX3330, is being tested in cancer clinical trials and could be applied to MPNST.

**Methods:**

We characterised Ref-1 and p-STAT3 expression in various MPNST models. Tumour growth, as well as biomarkers of apoptosis and signalling pathways, were measured by qPCR and western blot following treatment with inhibitors of Ref-1 or STAT3.

**Results:**

MPNSTs from *Nf1-Arf*^*flox/flox*^*PostnCre* mice exhibit significantly increased positivity of p-STAT3 and Ref-1 expression when malignant transformation occurs. Inhibition of Ref-1 or STAT3 impairs MPNST growth in vitro and in vivo and induces apoptosis. Genes highly expressed in MPNST patients are downregulated following inhibition of Ref-1 or STAT3. Several biomarkers downstream of Ref-1 or STAT3 were also downregulated following Ref-1 or STAT3 inhibition.

**Conclusions:**

Our findings implicate a unique therapeutic approach to target important MPNST signalling nodes in sarcomas using new first-in-class small molecules for potential translation to the clinic.

## Background

Malignant peripheral nerve sheath tumour (MPNST) is a rare soft-tissue sarcoma that can arise in patients with neurofibromatosis type 1 (NF1). These patients are at a significantly increased risk of developing MPNST than the general population (10% vs 0.01%, respectively). The development and pathogenesis of these tumours are still under investigation, but preclinical studies and mouse models are aiding in this discovery. An essential characteristic of NF-deleted tissues that have the propensity to progress to malignant tumours is the benign peripheral nerve sheath tumours called plexiform neurofibromas (PN). MPNST can arise from PNs and is believed to progress from PN to atypical neurofibromatous neoplasms of uncertain biologic potential (ANNUBP, formerly referred to as atypical neurofibroma) to MPNST.^1^ Despite ongoing research, existing chemotherapeutic and targeted agents have had limited success in MPNST treatment with a 5-year patient survival rate of just 35–50%.^2^

The loss of the *NF1* gene and subsequent protein product, neurofibromin, results in constitutive activation of Ras signalling and an increase in proliferative capacity. In KRAS-driven cancers, there is crosstalk between many signalling networks, including KRAS, PDGFR and MET, and many of these pathways are upstream of STAT3. This provides the tumour with a growth advantage making them difficult to treat and often inherently resistant to treatment.^3,4^ Recent research implicates signal transducer and activator of transcription-3 (STAT3) and hypoxia-inducible factor 1 (HIF1-α) in driving MPNST,^5^ and knockdown or pharmacological inhibition of STAT3 or HIF1-α inhibited tumour growth.^6,7^ Both STAT3 and HIF1-α are transcription factors implicated in signalling in many important cancer-related pathways and respond to a variety of tumour-related stress including hypoxia and inflammatory signalling.^8^ Activated STAT3 (p-STAT3) indicates aggressive disease at disease onset in MPNST.^5,9,10^ STAT3 can be activated through cytokines and growth factors. IL-6 stimulates phosphorylation of STAT3 leading to dimerisation and translocation to the nucleus to activate gene transcription.^11^ Expression of p-STAT3 is higher in MPNST compared to neurofibromas or Schwannomas, and there was a strong correlation between prolonged survival and low expression of p-STAT3.^9^ These data support STAT3 as a target in MPNST to potentially slow the growth of the tumour. Both HIF1-α and STAT3 have been inherently difficult to target with specific inhibitors.^12^ To circumvent this targeting difficulty, we have focused on the protein, Redox effector factor-1 (Ref-1) that interacts and regulates STAT3 and HIF1-α DNA binding and subsequently activates transcription.^13–15^ Based on the importance of these two transcription factors in other MPNST studies, as well as multiple studies showing high expression levels of Ref-1 indicating decreased survival in a number of cancers,^8^ we investigated the impact of targeting either Ref-1 or STAT3 in MPNST cells.

Ref-1 is a multifunctional protein in redox regulation of various transcription factors, including HIF1-α, NFκB and STAT3.^16^ It is also known to act as an apurinic/apyrimidinic endonuclease repairing DNA damage as well as reported functions in RNA processing, and interactions with nucleophosmin 1 (NPM1).^16^ We characterised Ref-1 and pSTAT3 protein levels in MPNST patient samples from the IN Pediatric BioBank, in established MPNST cell lines, and in a new patient-derived tumour xenoline (RHT-92). Ref-1 and pSTAT3 were also characterised in the *Nf1-Arf* ^*flox/flox*^*;PostnCre* mice that harbour combined genetic inactivation of *Nf1* and the *Cdkn2a* alternate reading frame (*Arf*), which is deleted in 70–90% of NF1-associated ANNUBP and MPNST collectively.^17^ These mice spontaneously develop nerve sheath tumours histopathologically indistinguishable from human ANNUBP and which progress to MPNST with high penetrance. Malignant transformation of plexiform neurofibroma and ANNUBP precursor tumours to MPNST was associated with marked upregulation of p-STAT3 and Ref-1 in these genetically engineered mice. Collectively, these data indicate that p-STAT3 and Ref-1 are highly expressed in both murine and human MPNST and further support their importance in this disease.

Knockdown of Ref-1 or STAT3 impairs MPNST growth in vitro, supporting the role of these genes in MPNST survival. However, a more clinical approach using small molecules to inhibit the redox activity of Ref-1 utilised Ref-1-specific inhibitors, APX3330 (and next-generation analogues, APX2009 and APX2014) and this treatment blocked in vitro cell proliferation. Likewise, inhibiting STAT3 activity using Ruxolitinib (Rux) or Napabucasin (Napa) also resulted in reduced in vitro cell proliferation.^18–20^ APX3330 has recently completed Phase 1 clinical trial in solid tumours with strong data supporting target engagement with reduced levels of transcription activity of STAT3, as well as NFκB and HIF1-α. In addition, a decrease in Ref-1 serum levels and circulating tumour cells was observed. Six of the 19 patients on the trial had disease stabilisation for over 12 weeks and four for over 36 weeks. APX3330 was well tolerated and an RP2D (recommended Phase 2 dose) obtained.^21^ Analogues of APX3330 including APX2009 and APX2014 have been developed from vigorous SAR activity and demonstrate similar safe toxicity profiles as APX3330 with increased efficacy in multiple models.^20,22^

Mechanistically, we investigated several means of cell death after inhibition of Ref-1 and/or STAT3, including reactive oxygen species (ROS) generation, PARP cleavage and caspase-3 activity. We also confirmed inhibition of Ref-1 and/or STAT3 using biomarkers downstream of Ref-1 and STAT3. Using previously published RNA-seq data that identified differentially expressed genes (DEGs) following Ref-1 knockdown^23^ and an independent gene expression dataset consisting of MPNST cell lines, NF1-derived neurofibroma Schwann cells and MPNST and NF1 tissue samples, we were able to identify genes that are upregulated specifically in MPNST,^24,25^ which can then be downregulated with inhibition of Ref-1 or STAT3 in MPNST. Combined with in vivo efficacy studies in mice treated with APX2009 or Napa, these results with clinically tested Ref-1 and STAT3 inhibitors make the future translation of this work highly plausible for paediatric patients with MPNST.

## Methods

### Cell culture

Immortalised human Schwann cell lines with mutant NF1 (ipNF05.5 and 95.6) and wt NF1 (ipn02.3) as well as malignant peripheral nerve sheath tumour (MPNST) cell lines ST88-14, S462 and NF90-8 were maintained at 37 °C in 5% CO_2_ and grown in Dulbecco’s Modified Eagle’s Medium (DMEM, Invitrogen, Carlsbad, CA, USA) with 10% Foetal Bovine Serum (FBS, Atlanta Biologicals, Minneapolis, MN, USA).^26^ Cells were authenticated by STR analysis and tested for mycoplasma contamination. ST88-14 and S462 were received from Dr. Andrew Tee (Cardiff University). MPNST cell line NF90-8 was received from Dr. Verena Staedtke (Johns Hopkins University).

Following resection, patient tissue (18-year-old female) was initially expanded as flank tumours, harvested and expanded as a cell line, which were referred to as ‘RHT-92’. Once the cells were growing in culture, we characterised them for MPNST markers as well as STR analysis (Supplementary Fig. 1). RHT-92 PDX cell line was checked for mycoplasma and confirmed negative prior to cryopreservation.

Cell proliferation and viability were measured with Alamar Blue assay as previously described.^14^ Briefly, ST88-14 and S462 cells were seeded at 1000 cells per well, and NF90-8 and RHT-92 cells were seeded at 2000 cells per well. Viability was measured 72 h after drug treatment. For the proliferation assays after siRNA transfections, conditions were optimised for each cell line for >80% knockdown of Ref-1 or STAT3. The NF90-8 cells were assayed on day 6 following transfection for both Ref-1 and STAT3 effects on proliferation, and ST88-14 cells were assayed on day 3. S462 cells were assayed on day 3 for the STAT3 knockdown and day 5 for Ref-1 (Fig. 2d).

### Immunohistochemistry

Tissue processing for wild-type (wt), Nf1^flox/flox-^;Postn-Cre and Nf1-Arf^flox/flox^;Postn-Cre mouse tissues was performed, as previously described.^17^ After euthanasia, mouse tissues were fixed in 10% neutral buffered formalin (NBF). After fixation, tissues containing bone were decalcified in 5% formic acid in NBF, and nerves were microdissected and transferred to 70% ethanol. Nerves were embedded in 5% agar prior to processing. All tissues were processed through graded alcohols, cleared in xylenes, infiltrated with molten paraffin and then embedded in paraffin blocks. Five-micron thick sections were cut and mounted on slides for staining.

Slides from MPNST patients were obtained through the Indiana Pediatric Biobank (IRB #1501467439) with informed patient consent and HIPAA compliance protocol. The human and GEMM slides were stained for Ref-1 (mouse Ref-1 antibody, 1:200 dilution, Novus Biologicals, 13B8E5C2) or p-STAT3 (Y705) (rabbit monoclonal 1:25 dilution, Cell Signaling, D3A7) in the Indiana University School of Medicine Research Immunohistochemistry Facility, as previously described.^13^ Stained slides were scanned with an Aperio CS2 Scanscope to generate whole-slide images. The tissues of interest were annotated and HALO (Indica Labs) or Aperio (Leica Biosystems, Wetzlar, Germany) Image Analysis software was used to identify the positive cells (HALO) or positive pixels (Aperio) for the immunohistochemistry chromogen, diaminobenzidine (DAB) substrate, by colour and optic density. HALO analysis of p-STAT3 and Ref-1 expression in mouse nerve tissue was generated by identifying cell nuclei with RBG (0.346, 0.317, 0.146), nuclear contrast threshold (0.589) and minimum nuclear OD (0.227). To properly identify cells of MPNST tissue, nuclear contrast was reduced to 0.535 and minimum nuclear OD to 0.088. Positive staining was determined with RGB values (0.452, 0.628, 0.716) and a minimum OD of 0.287. The percent positive cells were then calculated for each region of interest and averaged within each tissue type. For the Aperio Image analysis of Ki67 and pH3, percent positive pixels were calculated using the weak, moderate and strong staining with respect to the total number analysed for each annotation and averaged within each tissue type.

### siRNA transfections

MPNST cells were transfected with Ref-1 #1 siRNA (sequence previously reported^14,23,27^), LifeTech validated Ref-1 siRNA (s1447, Santa Cruz, Dallas, TX, USA), validated STAT3 siRNAs (sc-44275, sc-29493) and scrambled control. Ref-1 siRNA #1 and STAT3 siRNA sc-44275 were used as the standard siRNAs unless otherwise specified. Conditions were optimised for each cell line for the amount of siRNA and time point that corresponds to >80% knockdown: 30 nM of siRNA was used for ST88-14 and S462 cells, and 10 nM of siRNA was used for NF90.8.

### Inhibitors

Small-molecule Ref-1 inhibitors APX3330, APX2009 and APX2014 (Apexian Pharmaceuticals, Indianapolis, IN, USA) were prepared and used, as previously described.^13,20,23^ STAT3 inhibitor Napabucasin (SelleckChem, Houston, TX, USA) was dissolved in 100% DMSO and stored as a 20 mM stock at −20 °C. JAK1/2 kinase inhibitor Ruxolitinib (SelleckChem) was dissolved in 100% DMSO and stored as a 50 mM stock at −20 °C.

### Tumour spheroid assays

Two-layered soft agar assays were carried out in 6-well plates. MPNST cell lines were plated in complete DMEM media in 0.35% (w/v) agar at (6000 cells per well) over a 0.6% (w/v) agar layer. The agar was then overlaid with complete DMEM media and MPNST spheroids were grown for 14 days at 37 °C in 5% CO_2_. Media was changed twice a week in the presence or absence of drugs. Pictures were taken using an inverted AMG EVOS microscope equipped with an Olympus camera. The volume of tumour spheroids was measured using ImageJ (v1.48) software.

### Western blot analysis

Western blots were performed as previously described, with antibodies for Ref-1 (mouse, 13B8E5C2, Novus Biologicals, Centennial, CO, USA), HIF1-α (rabbit, GTX127309, GeneTex, Irvine, CA, USA), p-STAT3 (Y705, rabbit, D3A7), STAT3 (mouse, 124H6), Cleaved PARP (poly-ADP ribose polymerase, rabbit, D64E10), total PARP (rabbit, #9542) (Cell Signaling, Danvers, MA, USA), Vinculin (mouse, V284, Sigma, St. Louis, MO, USA) and Actin (mouse, ACTN05(C4), NeoMarkers) at 1:1000 dilution.^13–15,28^ For the HIF westerns, a urea lysis buffer (Tris, pH 6.8, 20 mM; NaH_2_PO_4_, 100 mM; urea, 6 M) was used for protein extraction.

### Wound-healing assay

ST88-14 and S462 cell lines were seeded in 35-mm plates. At 90% confluency, cells were starved (1% FBS) for 24 h. Each plate was then scratched using a pipette tip, and pictures were taken (0 h). ST88-14 and S462 cells were then treated with APX3330 at 50 µM and placed in the incubator (5% CO_2_/37 °C). After 18 h, the scratched area was measured using ImageJ, and the percentage of wound closure was calculated. A one-way ANOVA was performed using Prism to determine significance. Scale bar represents 250 µm. The change in the area of the scratch from 0 h to 18 h was imaged on a microscope at ×10 magnification and plotted as % distance migrated. Three individual experiments were performed.

### Intracellular ROS assays

ST88-14 was seeded at 16,000 cells/well in tissue culture-treated 96-well plates to assure 80–90% confluency for the ROS assay. Cells were treated with Ref-1 redox inhibitors, APX3330, APX2009 and APX2014 (Apexian Pharmaceuticals) and vehicle control (DMSO), all prepared in Opti-MEM (Gibco, Waltham, MA, USA) and treated for 2 h at 37 °C, 5% CO_2_. After treatment, ROS fluorescent detector CellROX^®^ Green Reagent (Molecular Probes, Eugene, OR, USA) was prepared in Opti-MEM at a concentration of 10 μM and then added to the drug media to a final concentration 5 μM and incubated with a reagent for 30 min. After incubation, the medium was removed, and the cells were gently washed three times with PBS. Cells were fixed with 3.7% formaldehyde for 15 min and then exchanged with PBS. ROS fluorescence was detected at 485/528 excitation/emission (BioTek Synergy H4, Winooski, VT, USA). The test was performed in three separate experiments and analysed by both Student’s *t* test and one-way ANOVA in Prism (GraphPad).

### Apoptosis assays

Annexin-V/PI staining: MPNST cells were plated and treated with Napa or APX2009 for the indicated time period and then assayed for apoptosis using Annexin-V Apoptosis detection Kit APC (Thermo Fisher) using flow cytometry.^15^

Caspase 3/7 activity via Incucyte system: NF90.8 and ST88-14 cells were plated in 96-well plates at 2500 cell/well and allowed to attach and grow for 48 h after plating. Increasing amounts of APX2009 or Napa were added to each well along with 1 μM of the caspase reagent (Caspase-3/7 Red, Essen Bioscience) and then the cells were allowed to recover for 2 h prior to beginning imaging with the Incucyte system (Essen Bioscience, Ann Arbor, MI, USA). Each well was imaged for phase contrast as well as red fluorescence every 2 h for 96 h. The Incucyte software-generated real-time imaging data and the red fluorescence was normalised to the percent confluency of each well and then to Media at time 0 as previously published.^22^

### RNA isolation, reverse transcription and real-time quantitative PCR (qRT-PCR)

Cells under indicated experiments were collected and processed for RNA extraction according to the manufacturer’s protocol (Qiagen, Hilden, Germany, USA). The RNA concentration was determined using a NanoDrop (Thermo Fisher). Subsequently, 1 μg of RNA/25-µl reaction mix was reverse-transcribed to cDNA using (Applied Biosystems). qRT-PCR was performed in 96-well plates, with a final volume of 20 μL/well using the SYBR Green PCR kit (Applied Biosystems, Foster City, CA, USA) on the CFX96 real-time PCR detection system (BioRad, Hercules, CA, USA). Primers for indicated genes are commercially available (OriGene, Rockville, MD, USA). qRT-PCR cycling conditions were 1 min at 95 °C, 10 min at 95 °C, 15 s at 95 °C and 1 min at 60 °C for 40 cycles. Relative changes in mRNA expression levels were assessed by the 2-ΔΔCT method and changes in mRNA expression of the target gene were normalised to that of β-actin gene.

### Bioinformatic analysis of transcriptomics data of MPNST genes

We have conducted a series of bulk and single-cell transcriptomic experiments after knockdown of Ref-1, including patient-derived cell lines of pancreatic cancer^23,29^ and MPNST cell lines (unpublished data). We further identified if any of the genes affected by Ref-1 knockdown identified in our previous study were upregulated in MPNST and could be possibly suppressed by inhibiting Ref-1.^29^ An independent gene expression dataset from GEO (https://www.ncbi.nlm.nih.gov/geo/query/acc.cgi?acc=GSE14038), consisting 13 MPNST cell lines, 11 plexiform NF1 cell lines, six MPNST and 13 NF1 tissue samples, was utilised to identify genes that are upregulated in MPNST compared to the benign neurofibromas.^24,25^ Thirteen genes that were highly upregulated in MPNST compared to NF1-derived neurofibroma Schwann cells (all with FDR < 0.05 by Mann–Whitney test) and also affected by Ref-1 knockdown experiments were selected for further experimental validation.^30^

### In vivo studies

NOD.Cg-Prkdc^scid^ Il2rg^tm1Wjl^/SzJ (NSG) male mice (6–8 weeks of age) were obtained from the on-site breeding colony of the In Vivo Therapeutics Core of the Indiana University Simon Cancer Center. Animals were maintained under pathogen-free conditions and maintained on Teklad Lab Animal Diet (TD 2014, Harlan Laboratories USA) with ad libitum access to sterile tap water under a 12-h light–dark cycle at 22–24 °C. MPNST cell line ST88-14 was grown in culture and harvested for the subcutaneous implant (5 × 10^6^ cells/mouse) in the flank of NSG mice. Prior to treatment, we ensured that the tumours were in log-phase growth by waiting until the tumour volume reached 40–50 mm^3^ and increased in volume by approximately twofold over the next 3 days. The animals were then randomised based on tumour size using matched distribution analysis from Studylog system software (Pacifica, CA, USA) and treated with 35 mg/kg APX2009 (BID, IP, *n* = 11), 75 mg/kg Napa (BID, PO, *n* = 9) or vehicle (*n* = 9–10). Vehicle for the APX2009 was Propylene Glycol, Kolliphor HS15, Tween 80 (PKT) as previously reported^27^ and the vehicle for Napa was methylcellulose with 5 days on, 2 days off schedule for 3 weeks. Tumour tissues were harvested, fixed in formalin and processed for histological analysis, and then analysed for proliferation markers, pH3 and Ki67. The method of euthanasia was CO_2_ inhalation with the secondary (confirmatory) method of cervical dislocation. All procedures were approved by the Institutional Animal Care and Use Committee at the IU School of Medicine.

### Statistics

All the experiments were performed at least three independent times. The data obtained were expressed as ‘Average + Standard Error’. Significance was calculated as per either two-way ANOVA or unpaired *t* test wherever applicable using GraphPad Prism Version 8. For qRT-PCR, analysis of covariance models was performed to test the Ct difference of each target gene value between treatment with APX2009, Napa and vehicle (DMSO) or siRNA and scrambled control after standardisation by reference gene (Actin) using analysis of covariance (ANCOVA) as previously described.^31^ A *P* value of at least < 0.05 was considered statistically significant. For in vivo experiments, power calculations give 80% power to detect a significant difference as small as 6.20 (effect size 1.2) in mean tumour size. The type I error rate is 5%. Power projections used to generate these numbers indicate that the proposed sample size is adequate to test the effects anticipated by our pilot data.

## Results

### Ref-1 and STAT3 were expressed in PN, ANNUBP and MPNST patient samples

**Fig. 1:**
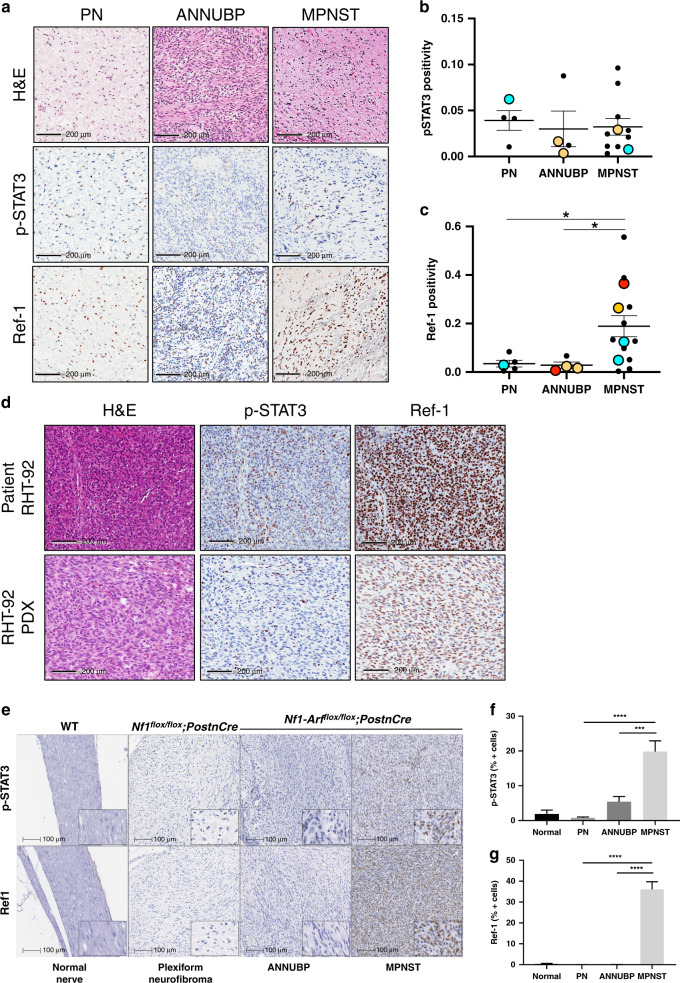
Expression of Ref-1 and p-STAT3 in tissue from patient samples and in mice that have conditional ablation in the Schwann cells of Nf1^−/−^; Arf^+/−^ and Nf1^–/–^; Arf^−/−^. IHC for p-STAT3 and Ref-1 levels in PN (*n* = 4), ANNUBP (*n* = 4) and MPNST patient samples (*n* = 14) with representative IHC images in (**a**) and quantitation by Aperio for pixel positivity (**b**, **c**, *P < 0.05) where the large circles with the same colour correspond to a sample from the same patient. (**d)** pSTAT3 and Ref-1 IHC images for MPNST patient sample and its corresponding PDX. Tissue from genetically engineered mouse models (**e**) was analysed by HALO for positive cells (**f**, **g**). For panels **a** and **e**, representative pictures are shown (**e**, *n* = 4, ***P < 0.001, ****P < 0.0001). Scale bar: 100 or 200 μm as noted.

We obtained PN, ANNUBP and MPNST patient samples from the Indiana Pediatric Biobank to quantify Ref-1 and p-STAT3 protein expression (Fig. 1a–c). The MPNST samples had a range of Ref-1 expression with significantly more Ref-1 expression than PN or ANNUBP (Fig. 1c). Figure 1a, b also illustrates p-STAT3 staining in all three groups with similar expression levels between them (Fig. 1b). This antibody is specific to the Y705 residue and is indicative of activated STAT3. Using tumour tissue from Patient #1 passaged in NSG mice, a xenoline was established (referred to as RHT-92). IHC markers on the PDX were compared to the original tumour. The PDX tumour retained positivity for Ref-1, p-STAT3 and PGP9.5 and retained lack of expression of S100 and CD56 (Fig. 1d and Supplementary Fig. 1), confirming retention of key IHC markers from the original tumour.

Nf1-Arf^flox/flox^;PostnCre mice exhibited high expression of Ref-1 and p-STAT3 in MPNST as compared to plexiform and atypical neurofibroma precursor tumours. We examined the expression of p-STAT3 and Ref-1 in mice that have conditional ablation *Nf1* and *Arf* in neural crest-derived Schwann cells that spontaneously develop a full spectrum of NF1-associated nerve sheath tumours including plexiform and atypical neurofibroma as well as MPNST. We found that malignant transformation of plexiform neurofibroma and ANNUBP precursor tumours to MPNST was associated with significant upregulation of p-STAT3 and Ref-1 in these genetically engineered mice (Fig. 1e–g). The increase in expression of p-STAT3 appeared to correlate with disease progression (Fig. 1e, f). The observed increase in this GEMM model confirmed a study comparing human MPNST to neurofibromas in which p-STAT3 also appeared to play a role in the progression of the disease,^9^ but was in contrast to our data in human samples where the p-STAT3 levels remained constant. In contrast, increases in Ref-1 were restricted mainly to the malignant transformation matching the pattern in the human samples in Fig. 1c (Fig. 1g) (*P* < 0.0001). These data demonstrated the presence of both activated STAT3 (pY705) and Ref-1 in patient samples and a clinically relevant mouse model with changes in Ref-1 expression within the malignant phenotype in the mouse model and in the human samples. The data from the human samples demonstrated more variability than the genetically engineered mouse model. Both Ref-1 and p-STAT3 are highly expressed in MPNST samples, which led us to hypothesise that targeting of these pathways may be therapeutically relevant in MPNST.

### Human Schwann cells and MPNST cell lines expressed Ref-1 and STAT3 and knockdown of these genes resulted in reduced proliferation in MPNST lines

**Fig. 2:**
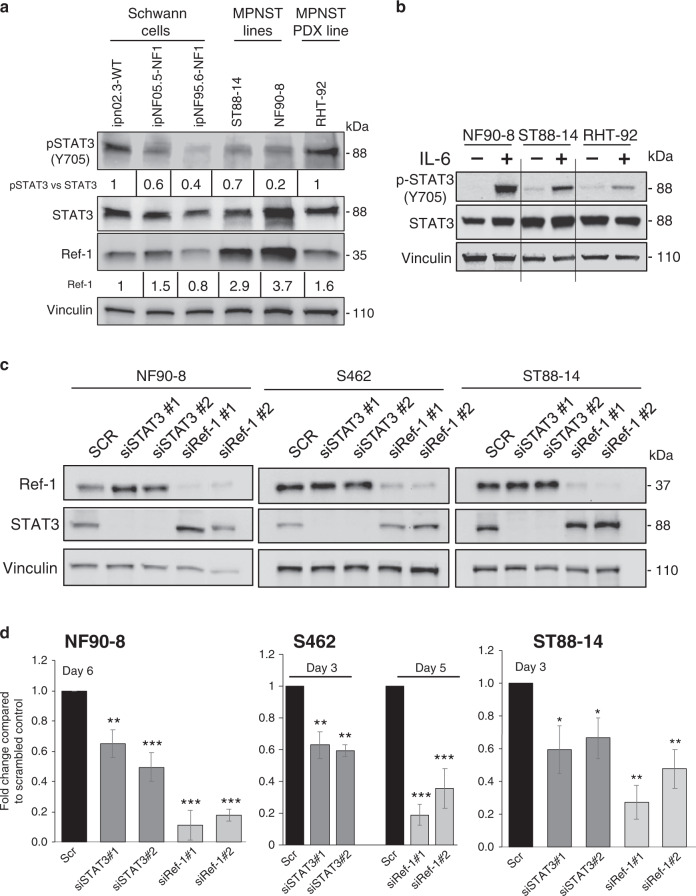
Human Schwann and MPNST cell lines express Ref-1 and STAT3 and a reduction in the protein levels of both proteins reduced cellular proliferation in MPNST lines. Western blot demonstrating the levels of p-STAT3 (Y705) and Ref-1 protein in wt and NF1-immortalised Schwann cells and in MPNST cell lines (**a**). Expression of Ref-1 in wt Schwann cells was set to 1 in order to compare the levels between lines. p-STAT3 levels were normalised to total STAT3 levels and then wt Schwann cells set to 1. (**b)** Expression of STAT3 and activated p-STAT3 after stimulation with IL-6 at 50 ng/mL for 15 min in NF90-8, ST88-14 and RHT-92 lines. Representative western blots are shown. Densitometry for fold change was done using Image Lab software from BioRad. (**c)** Knockdown of Ref-1 or STAT3 in various MPNST cell lines by at least 85% results in a decrease in cell proliferation by Alamar blue assay (**d**, **P < 0.01, ***P < 0.001, an average of four to six experiments ± SE, Student’s *t* test compared to scrambled control).

Figure 2a demonstrates the protein levels of Ref-1 in immortalised Schwann cells (wt and NF1), two MPNST cell lines and the xenoline RHT-92. For comparison of Ref-1 levels, the immortalised wt Schwann cells were used as the baseline. The NF1 Schwann cells had similar Ref-1 expression to the wt cells, while the MPNST lines were upregulated approximately twofold to threefold. Similarly, these cells expressed STAT3 protein with some basal level of STAT3 activation in wt, ipNF05.5, ST88-14, NF90-8 and RHT-92 cells, as assessed with the phosphorylation-specific antibody to the Tyr 705 residue. The NF1 Schwann cells and the MPNST lines had lower levels of STAT3 activation than the wt Schwann cells. These data match the patient data in Fig. 1b, c in which the levels of p-STAT3 are similar between benign and tumour cells, while the Ref-1 levels are higher in the tumour cell lines.

The MPNST cells’ response to STAT3 pathway activator, IL-6 and hypoxia was also assessed (Fig. 2b and Supplementary Fig. 2). NF90-8, ST88-14 and RHT-92 all responded to IL-6 stimulation as seen by the 17–300-fold increase in Y705 phosphorylation. However, S462 cells did not activate STAT3 in response to IL-6 and had lower levels of total STAT3 in comparison to ST88-14 and NF90-8 cells (Supplementary Fig. 2A). ST88-14, NF90-8 and S462 demonstrated stabilisation of HIF1 upon exposure to 1% hypoxia for 24 h (Supplementary Fig. 2B).

To study the effects of Ref-1 and STAT3 expression on MPNST cell proliferation in vitro, we utilised two separate siRNAs to reduce protein expression. In Fig. 2c, NF90-8, S462 and ST888-14 human MPNST cells were treated with STAT3 and Ref-1 siRNA, resulting in a reduction in the amount of protein by >85% versus scrambled siRNA controls on day 3 post transfection. Vinculin was utilised as a protein-loading control. Knockdown of STAT3 and Ref-1 reduced proliferation of these cells and the effects on cell growth were more pronounced with Ref-1 siRNA compared to STAT3 siRNA (Fig. 2d).

### Pharmacologic inhibition of Ref-1 and STAT3 abrogated MPNST cell proliferation

**Fig. 3:**
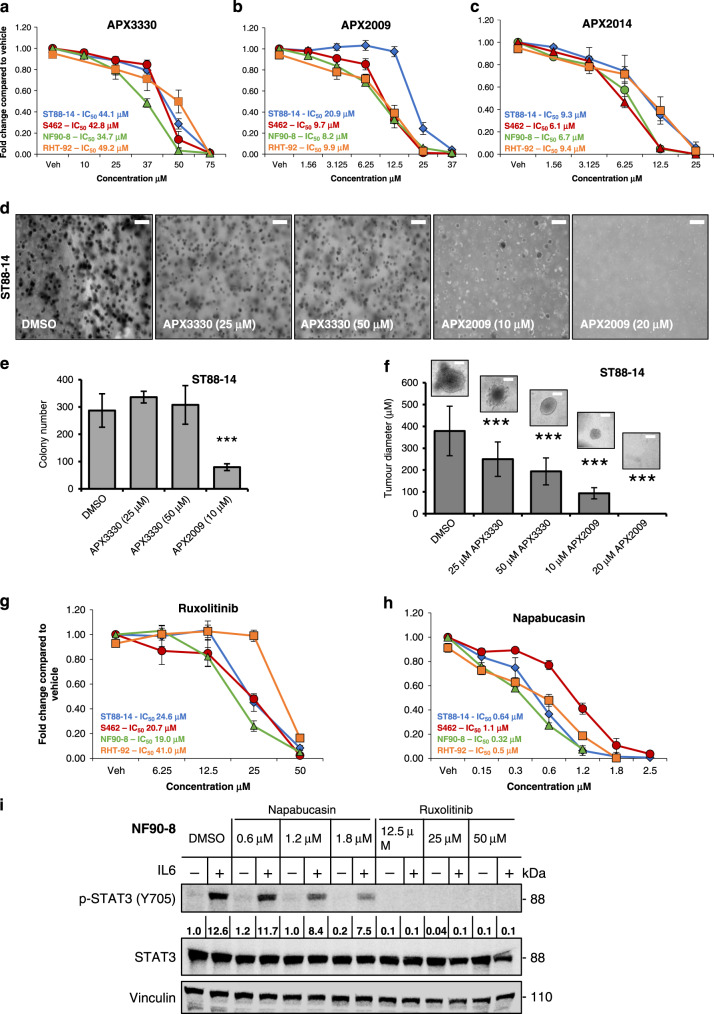
Ref-1 and STAT3 pathway inhibitors blocked proliferation and colony formation of MPNST cells. Alamar blue assay was used to determine dose response curves of various MPNST cell lines to Ref-1 inhibitors (**a–c**) with IC_50_s noted. Soft agar colony assays (**d**) demonstrate that Ref-1 inhibitors blocked the number of ST88-14 colonies formed (**e**; scale bar 0.5 mm) and resulted in smaller colonies (**f**, ***P < 0.001; scale bar 0.125 mm). Alamar blue assay following treatment with STAT3 inhibitors, Ruxolitinib (**g**) and Napa (**h**) in MPNST cells, *n* = 3 ± SE. Rux and Napa blocked the activation of STAT3 in NF90-8 cells as determined by western blot in (**i**).

Based on these results and the robust expression of both STAT3 and Ref-1 in MPNST cells and patient samples, we treated the tumour cells with small-molecule inhibitors of Ref-1 or STAT3 signalling. To inhibit Ref-1 redox activity, parent compound APX3330 that recently completed Phase 1 clinical trial (NCT03375086)^21,32^ and the second-generation analogues APX2009 and APX2014 were utilised.^20,22^ Inhibition of Ref-1 redox activity significantly reduced MPNST cell proliferation in vitro (Fig. 3a–c). In addition to a reduction in proliferation, there was a significant block in cell migration. Wound closure assay indicated a 40–50% decrease in migration in ST88-14 and S462 cells after treatment with APX3330 (Supplemental Fig. 3A, B). Increased potency was observed with analogues, APX2009 and APX2014 in the panel of MPNST cell lines compared to APX3330 (3–7-fold decrease in IC_50_, Fig. 3a–c). Tumour spheroid assays with ST88-14 cells in soft agar confirmed the proliferation assay effects of APX3330 and APX2009. APX2009 was more potent and significantly reduced both colony number and size at fivefold reduction in dosage compared to APX3330 (Fig. 3d–f).

To block STAT3-mediated signalling, MPNST cells were treated with Ruxolitinib (Rux) or Napabucasin (Napa). There was a dose-dependent decrease in cell proliferation with both drugs, with Napa showing IC_50_s in the nanomolar range (Fig. 3g, h). The doses of Rux and Napa that demonstrated inhibition of cell growth corresponded to doses that inhibited pSTAT3 as shown in Fig. 3i. A reduction of at least 2-fold in phosphorylated STAT3 (Y705) was observed with both Rux and Napa in NF90-8 cells (Fig. 3i). MPNST cells were treated with APX2009 and Napa to determine whether the cells were undergoing apoptosis following treatment as well as an investigation into the blockade of signalling following treatment. Due to previous studies demonstrating in vivo efficacy with APX2009 and Napa and their potency in MPNST cells, these compounds were chosen for further evaluation of the mechanism behind the blockade of signalling in vitro and in vivo models.

### Blockade of Ref-1 or STAT3 signalling increased cell death via mechanisms of apoptosis involving activation of caspase 3 and 7

**Fig. 4:**
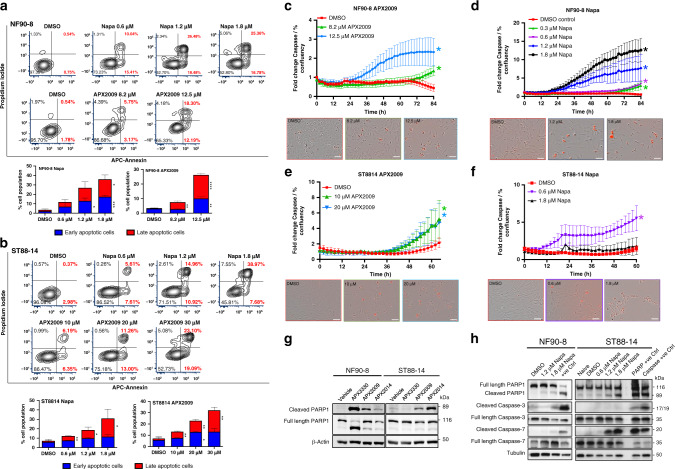
Induction of apoptosis in MPNST cells following treatment with Ref-1 and STAT3 inhibitors. Annexin-V/ PI staining of NF90.8 cells (**a**) treated with APX2009 (72 h) or Napa (48 h) at an indicated concentration (*n* = 3 ± SE; unpaired one-tailed *t* test *P < 0.05, **P < 0.01, ***P < 0.001). Annexin-V/PI staining of ST88-14 cells (**b**) after APX2009 or Napa treatment (48 h). The populations of early apoptotic cells (Annexin-V alone, blue) and the late apoptotic cells (Annexin-V and PI, red) are plotted in the graph in comparison to DMSO control (*n* = 3 ± SE, *P < 0.05, **P < 0.01). To monitor Caspase 3/7 activity, the Incucyte live imaging system was used in NF90-8 (**c**, **d**) and ST88-14 cells (**e**, **f**) after treatment with APX2009 (*n* = 4) and Napa (*P < 0.05, compared to the slope of vehicle control, *n* = 6). The increase in red fluorescence was normalised to % confluency. Western blots showing cleavage of PARP1, Caspase-3 and Caspase-7 in NF90-8 and ST88-14 cells following 48 h of treatment with Ref-1 inhibitors (**g**) and Napa (**h**, normalisation to total protein).

In order to determine whether the observed decrease in cell proliferation in Fig. 3 was due to cells undergoing apoptosis, Annexin-V/PI staining was used. As shown in Fig. 4a, b, the number of cells that were either in early apoptosis (Annexin V + , PI –) or late apoptosis (Annexin V + , PI + ) significantly increased when treated with APX2009 or Napa, and the effect was dose-dependent (*P* < 0.01). As a positive control for the apoptosis assay, NF90-8 and ST88-14 cells were treated with cisplatin that demonstrated Annexin V and PI positivity (Supplemental Fig. 4A, B).

To identify the molecular mechanisms of apoptosis following treatment, several assays were used, including Caspase-3 and -7 activation assay, PARP cleavage and ROS levels. We hypothesised that ROS generation may contribute to the mechanism of cell death following treatment with the APX compounds as well as Napa. Although significant, dose-dependent increases in ROS were observed following treatment, the increase in ROS was minimal with APX compounds as well as Napa and thus likely not a major contributor to the observed decrease in cell viability (Supplementary Fig. 4E).

Next, live-cell imaging with the Incucyte system was used to monitor confluency and activated Caspase-3 and -7, as previously described.^22^ Using the IC_50_ and IC_95_ of APX2009, we observed a significant dose- and time-dependent increase in cells that had active Caspase 3/7 as measured by red fluorescence normalised to cell confluency (Fig. 4c, e, *P < 0.05). Since both Caspase-3 and -7 can cleave PARP, we confirmed their activation using antibodies specific to cleaved and total PARP.^33^ Inhibition of Ref-1 resulted in an increase in PARP cleavage (Fig. 4g). APX3330 demonstrated activation of apoptosis via PARP cleavage (89 kD) with a dose of 60 μM in NF90-8, while APX2009 and APX2014 exhibited similar increases in cleaved PARP at three- to fivefold lower doses in NF90-8, ST88-14 and S462 cells compared to APX3330 (Fig. 4g and Supplementary 4C).

Similarly, Napa-induced Caspase 3/7 activation was evident in both NF90-8 and ST88-14 cells (Fig. 4d, f). The increase in fluorescence over time was dose-dependent and highly significant in the NF90-8 cells. In the ST88-14 cells, Napa decreased cell growth (as measured by confluency) at the IC_50_ dose (0.6 μM) and at the higher dose of 1.8 μM. While Caspase 3/7 activation was evident at the IC_50_ dose (0.6 μM), activation was not observed at the higher dose of Napa. As a positive control for activation of Caspase 3/7, ST88-14 cells were treated with Cisplatin, and strong activation was observed (Supplementary Fig. 4D). With Napa treatment, we observed an increase in PARP cleavage with the lethal doses as well as increases in cleaved Caspase-7 in ST88-14 cells and increases in both Caspase-3 and Caspase-7 cleavage in the NF90-8 cells (Fig. 4h). Combining all these data, we concluded that inhibition of Ref-1 with APX2009 and STAT3 with Napa resulted in cell death with increases in apoptotic cells as well as activation of apoptotic pathways via Caspase 3/7 and cleavage of PARP. The effects on the mechanisms of cell death of these compounds at high versus low doses may differ and remains an area of active study.

### Integrative analysis of RNA-seq following siRNA knockdown and published microarray data of human NF1 and MPNST revealed that key genes preferentially upregulated in MPNST were downregulated with APX2009 or Napa treatment

**Fig. 5:**
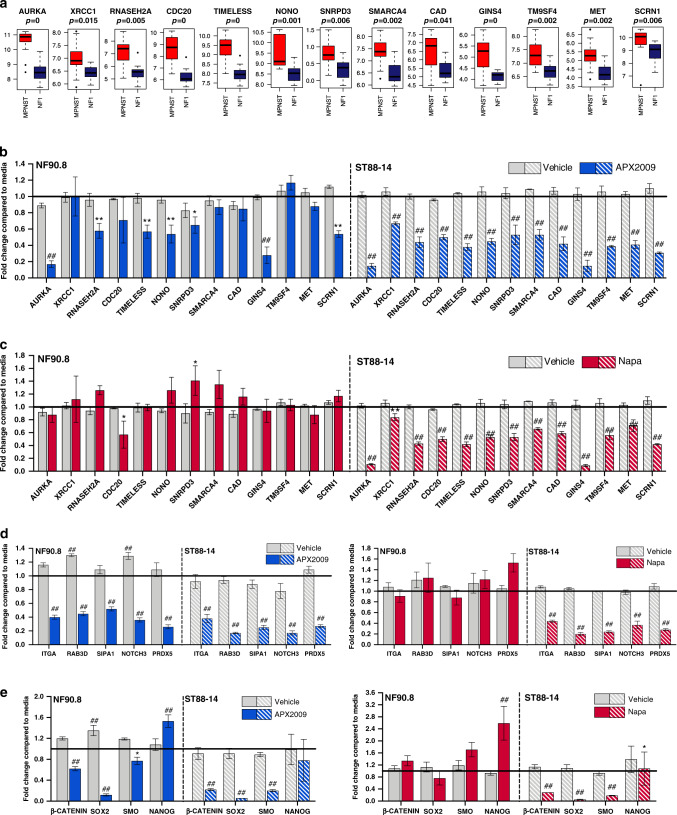
Ref-1 or STAT3 blockade downregulated expression of highly expressed genes in MPNST. A study from GEO database was used to discover genes that were more highly expressed in MPNST compared to benign NF1-derived neurofibroma Schwann cells. The *P* value indicated that the expression of these genes in MPNST cell lines is significantly higher than in NF1-derived neurofibroma Schwann cells. Boxplots show the comparison of the genes in MPNST (red) vs NF1-derived neurofibroma Schwann cells (dark blue) (**a**). qPCR evaluation of expression of 13-gene panels in ST88-14 cells following APX2009 treatment (20 μM, 24 h, **b**) or Napa treatment (1.6 μM, 24 h, **c**) and in NF90-8 cells (APX2009—25 μM, 24 h, **b** or Napa—1.8 μM, 24 h, **c**). qPCR was also used to evaluate genes indicative of Ref-1 inhibition (**d**) or genes that are biomarkers of Napa treatment (**e**) in ST88-14 and NF90-8 cells (*P* value ANCOVA model; *P < 0.05, **P < 0.01, ^##^*P* < 0.0001, *n* = 4–5).

To investigate genes that are differentially expressed in MPNST vs plexiform neurofibromas, we utilised a published microarray dataset with 13 human MPNST cell lines, 11 plexiform NF1 cell lines, six MPNST and 13 NF1 tissue samples from GEO database. We then correlated the differentially expressed genes (DEGs) in this dataset to the DEGs identified in our past transcriptomic analysis of Ref-1 knockdown.^23–25^ DEGs were identified that were downregulated in Ref-1 knockdown vs scrambled control in our published Ref-1 knockdown experiments, but upregulated in MPNST compared to benign samples. Specifically, 13 genes potentially regulated by Ref-1, namely AURKA, XRCC1, RNASEH2A, CDC20, TIMELESS, NONO, SNRPD3, SMARCA4, CAD, GINS4, TM9SF4, MET and SCRN1, were significantly upregulated in MPNST (Fig. 5a, *P* < 0.05).

Under conditions of both knockdown and pharmacological inhibition of Ref-1 or STAT3, the expression of these 13 genes was evaluated in ST88-14 and NF90-8 cells using qPCR (Fig. 5b, c and Supplementary Fig. 5A, D). Knockdown of Ref-1 via siRNA confirmed that the expression of these genes was significantly downregulated when Ref-1 activity was blocked in both NF90-8 and ST88-14 cells (Supplementary Fig. 5A, D). In ST88-14 cells, all 13 genes demonstrated a significant reduction in expression compared to scrambled control (Supplementary Fig. 5D). Although statistically significant, the MET gene was minimally downregulated in the NF90-8 cells (Supplementary Fig. 5A). This is in contrast to the ST88-14 cells in which MET expression is >70% reduced with Ref-1 knockdown. Next, we evaluated the expression of this 13-gene panel after treatment with Ref-1 inhibitor, APX2009 at a dose that corresponds approximately to IC_50_ after 72 h of treatment. The majority of these genes are also downregulated following treatment with APX2009 suggesting that Ref-1’s transcriptional regulator activity is responsible for the observed decrease in expression (Fig. 5b). In both NF90-8 and ST88-14 cells, AURKA, RNASEH2A, TIMELESS, NONO, SNRPD3, GINS4 and SCRN1 were downregulated by ~50% or more.

The effects of STAT3 knockdown on this 13-gene panel were less dramatic compared to the changes observed with Ref-1 (Supplemental Fig. 5A, D). After treatment with Napa in ST88-14 cells, 9/13 genes were downregulated ~50% (AURKA, RNASEH2A, CDC20, TIMELESS, NONO, SNRPD3, GINS4, TM9SF4 and SCRN1, P < 0.05, Fig. 5c); however, there were cell line differences as the NF90-8 only had significant downregulation with CDC20 following Napa treatment (*P* < 0.05, Fig. 5c). Interestingly, the effects of STAT3 knockdown and treatment with Napa were not as consistent in modulating this gene signature. The effects on gene expression following Napa treatment were more dramatic and significant than STAT3 knockdown, indicating that Napa may have other effects than STAT3 inhibition. However, the comparison of the knockdown and inhibitor data for Ref-1 in two MPNST cell lines demonstrated on-target effects of Ref-1 inhibitor, APX2009. These data support that Ref-1 inhibition and Napa treatment will potentially alter the expression of genes important in MPNST.

### Downregulation of pharmacodynamic (PD) markers following inhibition of Ref-1 and STAT3

In our previously published studies, we identified several genes (ITGA1, RAB3D, SIPA1, NOTCH3 and PRDX5) that were downregulated following Ref-1 knockdown or after treatment with APX3330, indicating that the redox activity of Ref-1 was mainly responsible for regulating the expression of these genes.^23^ To evaluate these markers of Ref-1 inhibition, NF90-8 and ST88-14 cells were transfected with Ref-1 and STAT3 siRNA (Supplemental Fig. 5B, E) or treated with APX2009 (Fig. 5d). These five genes were significantly downregulated with Ref-1 knockdown. However, only two (ITGA1, NOTCH3) out of the five were altered with STAT3 siRNA in ST88-14 cells and none of them in NF90-8 cells (Supplementary Fig. 5B, E). Similarly, APX2009 treatment resulted in downregulation of this five-gene panel in both NF90-8 and ST88-14 cells (Fig. 5d). The results on the Ref-1 PD markers with Napa treatment were not consistent between the two MPNST cell lines as downregulation was observed in ST88-14 but not in NF90-8 cells.

Last, NF90-8 and ST88-14 cells were evaluated for PD markers following treatment with Napa. Several reports in other solid tumours indicated that stemness or STAT3 target genes were decreased following Napa treatment, including β-catenin, Sox2, SMO and Nanog.^19,34^ We also investigated the expression of these genes under Ref-1 or STAT3 blockade. Significant downregulation of Sox2 was observed with APX2009 and Napa treatment as well as Ref-1 and STAT3 siRNA (Fig. 5d, e and Supplementary Fig. 5C, F). A reduction in the expression of β-catenin and SMO was more pronounced with the drug treatment compared to both siRNAs. Although decreased by ~30% with STAT3 siRNA, Nanog expression was not decreased with Napa treatment. The effects of Ref-1 siRNA and APX2009 treatment were not consistent on Nanog expression, as a 1.4-fold increase was observed with Ref-1 siRNA and no difference seen with inhibitor treatment. Thus, Nanog does not appear to be a reliable PD marker of treatment efficacy in MPNST (Fig. 5d, e and Supplementary Fig. 5C, F).

### In vivo treatment with APX2009 and Napa led to a reduction in tumour growth and proliferative markers

**Fig. 6:**
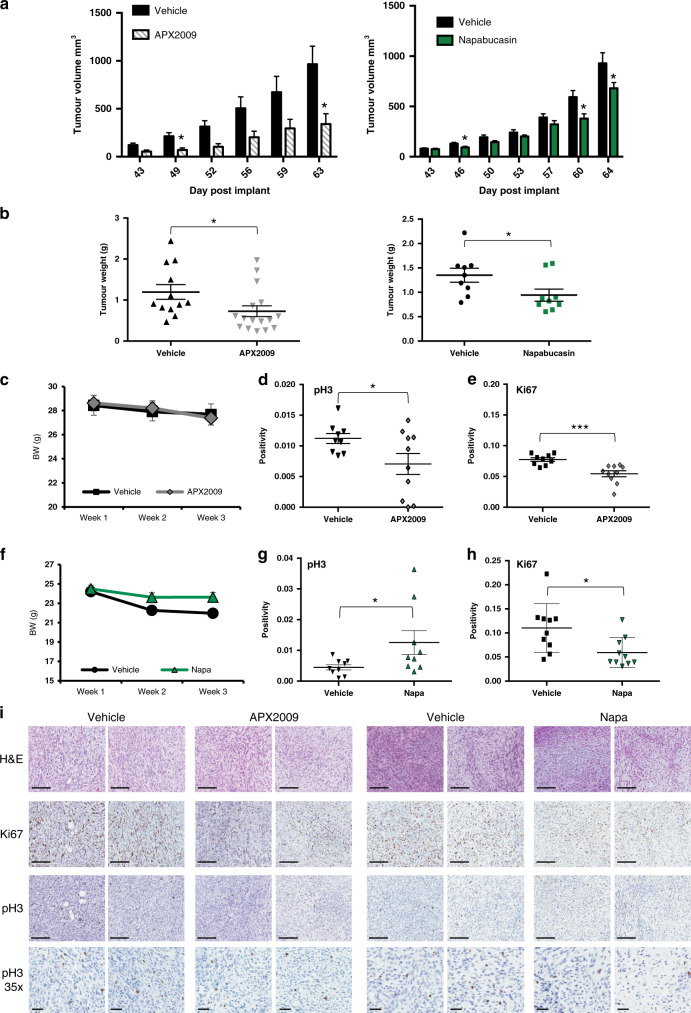
Treatment with APX2009 or Napa reduced the growth rate of MPNST tumours and proliferative capacity in vivo. Xenografted ST88-14 tumours treated with APX2009 (35 mg/kg) or Napa (75 mg/kg) resulted in slower-growing tumours (**a**, *P < 0.05) and a reduction in the size of the tumours at sacrifice (**b**, *P < 0.05). Neither treatment was overly toxic as the body weights of the treated mice were not different from the vehicle (**c**, **f**). IHC analysis of proliferative markers, pH3 (**d**, **g**; *P < 0.05) and Ki67 (**e**, **h**; *P < 0.05, ***P < 0.001) is shown with representative pictures in (**i)**. Scale bar is 200 μm unless otherwise indicated.

For the in vivo studies, ST88-14 cells were implanted subcutaneously into NSG mice and tumour growth was monitored over time. Both APX2009 (35 mg/kg) and Napa (75 mg/kg) decreased the tumour volume (Fig. 6a, *P* < 0.05) with a corresponding decrease in tumour weight at the end of study (Fig. 6b, *P* < 0.05). Although statistically significant, the effect on tumour growth following Napa treatment was not as robust as treatment with APX2009. Both dosing regimens were not overly toxic to the mice as body weights were maintained throughout the three weeks of dosing (Fig. 6c, f). IHC staining of tumours with proliferative markers, pH3 and Ki67 revealed expected decreases in proliferation in the APX2009-treated tumours with a 28% decrease in pH3 positivity and 3/10 tumours with limited detection of pH3 (Fig. 6d, i, *P* = 0.057). Ki67 positivity also decreased by ~30% in the APX2009-treated tumours (Fig. 6e, i, *P* < 0.001). In the Napa-treated tumours, the positivity of Ki67 was significantly decreased by 41% (Fig. 6h, i, *P* < 0.05); however, the pH3 positivity actually increased significantly (Fig. 6g, i, *P* = 0.055). This seemed to be driven by two tumours that scored higher than the others. The Ref-1 levels in the tumours following treatment with either APX2009 or Napa were unchanged (Supplementary Fig. 6). The results of these in vivo studies confirmed that APX2009 and Napa decrease tumour growth and reduce proliferation in treated tumours. The effects on tumour growth with APX2009 were more dramatic, supporting the rationale for Ref-1 signalling blockade in the treatment of MPNST.

## Discussion

MPNST is a rare, aggressive sarcoma that is highly refractory to conventional treatment modalities. An understanding of the critical signalling mechanisms in MPNST remains poor, hampering the development of effective targeted therapies. Several receptor tyrosine kinase signalling cascades, protein folding regulators, epigenetic regulators, Wnt pathway mediators and tumour microenvironment interactions have previously been implicated in modulating the development and progression of MPNST.^35^ Here, we demonstrate a pivotal role of the redox regulator Ref-1 and transcription factor STAT3 in driving MPNST cell survival and proliferation. Our previous studies demonstrated that STAT3 is under Ref-1 redox control in other cancers including pancreatic cancer.^15^ In addition, Rad et al.’s study in MPNST revealed the importance of STAT3 and HIF1-α in driving MPNST phenotype.^5^ Although STAT3 is regulated through Ref-1-dependent redox activity, there are several other forms of STAT3 regulation including phosphorylation and nuclear translocation. Inhibitors of Ref-1 (APX2009) and STAT3 (Napa) were used to test the efficacy of targeting these pathways in vitro and in vivo in human MPNST models. We found that STAT3 and Ref-1 are expressed in human Schwann cells, MPNST tumours and cell lines, as well as in MPNST that spontaneously develops from existing plexiform and atypical neurofibroma precursor lesions that harbour conditional ablation of *Nf1* and *Arf* in neural crest-derived Schwann cells. Intriguingly, in a powerful genetically engineered mouse model that develops MPNST, activated STAT3 emerges at the ANNUBP stage and then is significantly higher when these tumours undergo malignant transformation. Increases in Ref-1 expression, however, were predominantly restricted to MPNST as compared to precursor tumours (Fig. 1e, g). In MPNST cell lines, knockdown of Ref-1 with two different siRNA sequences dramatically reduced cell proliferation and was more pronounced than the effects on cell growth with siRNAs to STAT3 (Fig. 2d). Our previously published work demonstrated that STAT3 DNA binding could be enhanced by Ref-1 redox activity.^15^ However, Ref-1 can also activate many other transcription factors such as NFκB, p53 and HIF1-α. Figure 5b, c investigates the effects of APX2009 or Napa on the expression of a 13-gene panel that is upregulated in MPNST compared to NF1-derived neurofibroma Schwann cells. The on- or off-target effects of these small-molecule inhibitors were also compared to the knockdown of either Ref-1 or STAT3 (Fig. 5b, c and Supplementary Fig. 5A, D). There was much greater concordance with APX2009 and Ref-1 knockdown on gene expression than with Napa treatment and STAT3 knockdown. Six of these 22 genes are significantly downregulated by both Ref-1 and STAT3 siRNA, e.g., MET, ITGA1, Notch3, β-catenin, Sox2 and SMO in ST88-14 cells. Concurrent downregulation of these six genes implies that Ref-1’s activation of STAT3 is playing a role in their expression. The other genes are more dramatically affected by Ref-1 knockdown, but not STAT3 suggesting that Ref-1’s regulation of other transcription factors is driving the expression of these genes (Fig. 5).

Pharmacologic inhibition of Ref-1 and STAT3 abrogated the proliferative capacity of MPNST cell lines (Fig. 3). Ref-1 inhibitor, APX3330 is the parent compound and has recently completed Phase 1 clinical trials demonstrating a good safety profile, Ref-1 target engagement and some potential for therapeutic benefit.^21,32^ Two new analogues, APX2009 and APX2014 have increased potency with four- to fivefold increases in IC_50_ and dramatic effects on the colony formation in soft agar (Fig. 3a–f). Selective and specific STAT3 inhibitors have long been sought after due to STAT3’s role in stemness, invasion, metastasis and more.^28,36^ For these studies, two clinically relevant compounds were used: Ruxolitinib, a Jak1/2 inhibitor that blocks phosphorylation of STAT3, as well as Napa.^18,19,37,38^ The IC_50_s for Napa were sub-micromolar and were capable of inhibiting activated STAT3; therefore, we evaluated Napa as a novel therapeutic agent in MPNST as well as evaluating it as a STAT3 inhibitor by comparing the effects of gene expression following treatment to gene expression following knockdown of STAT3 (Supplementary Fig. 5).

To determine the mechanism of cell death following APX2009 or Napa treatment, Annexin-V/PI staining as well as cleavage of apoptosis-related proteins was used. Annexin-V/PI staining did indicate that a population of the cells were undergoing apoptosis following treatment with either inhibitor (Fig. 4a, b). However, this was not the entire story. Decreases in cell viability were concurrent with activation of caspase-3 and -7 as indicated from the Incucyte live-cell imaging (Fig. 4c–f). Western blotting also confirmed activation of caspase-mediated apoptosis with PARP1 cleavage and some cleavage of caspase 3 and 7 following Napa treatment, with cleavage of caspase-7 being more predominant than caspase 3, especially in ST88-14 cells (Fig. 4g, h). Interestingly, however, MPNST cells treated with 1.8 μM of Napa underwent death independent to caspase-3/7 activation (Fig. 4f). Necroptosis may be involved at higher doses of Napa, and therefore in the future, we will determine alternate forms of cleaved PARP as well as inhibit RIP kinase.^39,40^ Napa has been reported as a substrate for NAD(P)H dehydrogenase quinone 1 (NQO1) further supporting the role of alternate mechanisms of cell death.^18^ Napa may function as a substrate for NQO1 leading to the generation of superoxide and hyperactivation of PARP and eventually cell death.^40^ However, further experiments with multiple doses are needed for a clear conclusion.

Due to Ref-1 and STAT3’s role in regulating transcription, three sets of genes were evaluated by qPCR following siRNA and treatment with APX2009 and Napa. The first set of genes were generated by combining the differentially expressed genes (DEGs) from our published single-cell RNA-seq (scRNA-seq) data of Ref-1 knockdown^23^ and correlating these DEGs with genes that were significantly upregulated in MPNST samples compared to NF1-derived neurofibroma Schwann cells (Fig. 5). Validation of these genes with qRT-PCR using genetic or pharmacologic Ref-1 inhibition revealed interesting results. Notably, treatment with Ref-1 siRNA or APX2009 in human MPNST cell lines reduced the expression of these Ref-1 signature genes. These genes are involved in DNA replication or DNA repair (XRCC1, RNASEH2A and SCRN1), metabolism (CAD), protein folding and trafficking (TM9SF4), cell cycle (CDC20, GINS4) and circadian pathway (TIMELESS, NONO) (Fig. 5). The reduction in gene expression in Fig. 5b, c strongly supports Ref-1 as a target in MPNST, and these Ref-1-dependent genes are upregulated specifically in MPNST compared to NF1-derived neurofibroma Schwann cells. Due to previous studies demonstrating in vivo efficacy with APX2009,^20^ this compound was chosen for further evaluation in the in vitro and in vivo models.

This gene set was also evaluated with STAT3 siRNA as well as Napa treatment. Knockdown of STAT3 minimally, but significantly decreased the expression of RNASEH2A, CDC20, TIMELESS, SNRPD3, SMARCA4, CAD and GINS4, seven of the thirteen genes in both NF90-8 and ST88-14 lines. However, these genes were generated from RNA-seq data with Ref-1 knockdown; therefore, the lack of an effect on these genes with STAT3 siRNA was not surprising. In contrast, Napa treatment decreased all thirteen genes significantly, with the largest changes on AURKA and GINS4 (Fig. 5c). There is a lack of correlation between the STAT3 siRNA gene expression results and the Napa treatment.

The second set of genes that were tested were Ref-1 redox-associated genes from our previous study. This five-gene panel was downregulated when Ref-1 was knocked down or inhibited by APX3330 in pancreatic cancer xenolines.^23^ In MPNST cells, Ref-1 siRNA and treatment with APX2009 also significantly decreased the expression of these genes (Supplementary Fig. 5B, E and Fig. 5d). The effects of Ref-1 siRNA and APX20009 were similar with respect to gene expression that suggests on-target effects of the drug. STAT3 inhibition either with siRNA or Napa was not as consistent with respect to the Ref-1 redox-associated genes (Supplementary Fig. 5B, E and Fig. 5e). Napa treatment was able to significantly downregulate all five genes, while STAT3 siRNA did not change the expression of RAB3D, SIPA1 or PRDX5 significantly. Although inhibition of Ref-1 blocks the activity of STAT3, STAT3 is not the only transcription factor that Ref-1 can activate; therefore, the downregulation of the redox-associated genes may be due to AP-1 or NFκB inhibition rather than STAT3 (Fig. 5e). The results of the qPCR studies demonstrate that while some of the genes that exhibit decreased gene expression are similar with both STAT3 siRNA and Napa treatment, it is possible that some of the effects on the cell growth and gene expression by Napa may be off-target from STAT3.

The third set of genes (β-catenin, SOX2, SMO and Nanog) were chosen based on other published reports of PD markers conducted using Napa in other solid tumours.^19,34,41^ These hallmark stemness markers were also significantly decreased following Napa treatment in MPNST with the exception of Nanog (Fig. 5e, panel 2). Similarly, STAT3 siRNA also significantly decreased the expression of β-catenin, SOX2, SMO and Nanog (Supplementary Fig. 5C, F). Interestingly, knockdown of Ref-1 decreased the expression of these stemness markers: β-catenin, SOX2 and SMO, but not Nanog (which was slightly increased) (Supplementary Fig. 5C, F). This result was consistent with a decrease in the gene expression following treatment with APX2009 (Fig. 5d, panel 2). The gene expression patterns in Fig. 5 suggested that Napa has off-target effects in addition to hitting a subset of STAT3-regulated genes. However, it is important to emphasise that decreased expression of stemness genes and induction of apoptosis as previously reported still supports Napa as a potential therapeutic in MPNST. In general, there is good consensus on gene expression changes between Ref-1 siRNA and APX2009 treatment supporting that APX2009 is specifically targeting the redox activity of Ref-1.

In any single preclinical model, there are limitations to the application to human cancer. For future studies, additional in vivo models will be used to evaluate Ref-1 and STAT3 as targets in MPNST. The genetically engineered mouse model will be utilised to test whether blockade of STAT3 in earlier stages of NF1-associated nerve sheath tumour progression in plexiform neurofibroma and/or MPNST may have utility in MPNST chemoprevention. There are advantages and disadvantages of using mouse models in testing new therapeutics. In future studies, we would like to utilise the *Nf1-Arf* ^*flox/flox*^*;PostnCre* mice in addition to orthotopic PDX models. The GEMM model allows us to evaluate the disease in a model in which MPNST develops over time similar to that observed in patients. This GEMM has combined genetic inactivation of *Nf1* and the *Cdkn2a* alternate reading frame (*Arf*) that is deleted in 70–90% of NF1-associated ANNUBP and MPNST collectively^17^ and closely resembles patient samples. The Ref-1 expression pattern between the GEMM model and the human samples was strikingly similar, and yet in contrast, the p-STAT3 did not increase as the disease progressed as we observed in the GEMM. The orthotopic PDX model allows us to evaluate the effects of novel therapy on the growth of human MPNST samples that have not undergone laboratory manipulation. These GEMM and orthotopic studies are important as the work shown in Fig. 6 utilises only one cell line in vivo to test the effects of Ref-1 inhibition and Napa treatment. We hope that the two models together will provide collective insights for this highly aggressive cancer that has no effective therapies currently.

The use of STAT3 degraders or antisense oligos could provide an alternative to pharmacologic inhibitors that exhibit potential off-target effects. With the completion of the APX3330 clinical trial, the translation of this work highly plausible for paediatric patients with MPNST. Ref-1 also possesses a DNA repair function and is involved in RNA splicing, transport and stability; therefore, the knockdown of this protein could result in additional effects on cell survival. Consequently, the investigation into the effects of inhibition of Ref-1’s additional activities is hence a plan for our future research. Furthermore, we will use orthotopic and PDX models to look at relevant combination therapies to pair with Ref-1 or STAT3 inhibition. This work is another step towards validating a new target (redox factor-1, Ref-1) that is upstream of several pathways known to contribute to driving the disease including HIF1 and STAT3. Furthermore, preclinical validation for two novel therapeutic agents in this deadly cancer and future studies will pave the way for appropriate and rational combination therapies.

## Supplementary information

Supplemental Figures

## Data Availability

We used publicly available data to analyse the genes that were upregulated with MPNST compared to benign neurofibromas and that data can be found here (https://www.ncbi.nlm.nih.gov/geo/query/acc.cgi?acc=GSE14038). The previously published data that were used to determine Ref-1-regulated genes can be found in the GEO, accession number GSE99305. The RNA sequencing data in MPNST cells are currently being submitted to the GEO database.
