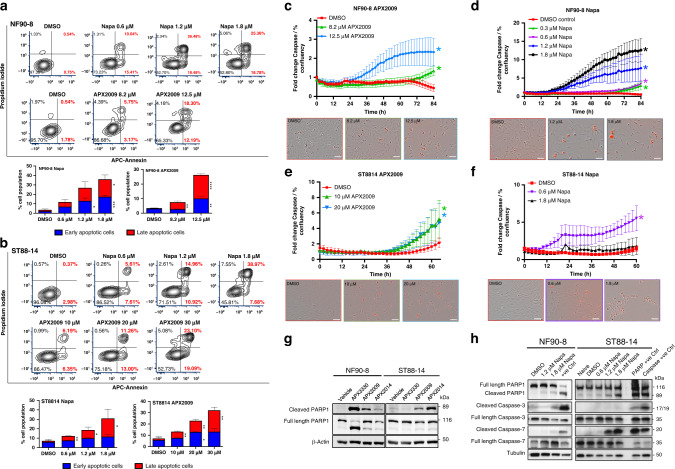# Correction: Exploring transcriptional regulators Ref-1 and STAT3 as therapeutic targets in malignant peripheral nerve sheath tumours

**DOI:** 10.1038/s41416-022-01938-9

**Published:** 2022-08-11

**Authors:** Silpa Gampala, Fenil Shah, Chi Zhang, Steven D. Rhodes, Olivia Babb, Michelle Grimard, Randall S. Wireman, Ellie Rad, Brian Calver, Ren-Yuan Bai, Verena Staedtke, Emily L. Hulsey, M. Reza Saadatzadeh, Karen E. Pollok, Yan Tong, Abbi E. Smith, D. Wade Clapp, Andrew R. Tee, Mark R. Kelley, Melissa L. Fishel

**Affiliations:** 1 Department of Pediatrics and Herman B Wells Center for Pediatric Research, Indiana University, School of Medicine, Indianapolis, IN USA; 2 Department of Medical and Molecular Genetics, Indiana University, School of Medicine, Indianapolis, IN USA; 3 Department of Electrical and Computer Engineering, Purdue University, West Lafayette, IN USA; 4 Division of Cancer and Genetics, Cardiff University, Cardiff, Wales UK; 5 Neurosurgery and Neurology, Johns Hopkins School of Medicine, Baltimore, MD USA; 6 Department of Pathology and Laboratory Medicine, Indiana University, School of Medicine, Indianapolis, IN USA; 7 Department of Pharmacology and Toxicology, Indiana University, School of Medicine, Indianapolis, IN USA; 8 Department of Biostatistics and Data Management, Indiana University, School of Medicine, Indianapolis, IN USA

**Keywords:** Paediatric cancer, Sarcoma

Correction to: *British Journal of Cancer* 10.1038/s41416-021-01270-8, published online 03 March 2021

The original version of this article unfortunately contained an error in Figure 4, specifically:

- Figure 4f: the middle cell image was originally a duplicate of the middle cell image from Fig. 4d; the correct image is now used.

The corrected figure is displayed below. The correction does not have any effect on the final conclusions of the paper. The original article has been corrected.