# Supplementary material for: Exploring transcriptional regulators Ref-1 and STAT3 as therapeutic targets in malignant peripheral nerve sheath tumours
Source: Br J Cancer. 2021 Mar 3;124(9):1566–80. doi: 10.1038/s41416-021-01270-8 (PMC8076291; doi:10.1038/s41416-021-01270-8)
Supplement: Supplementary file 1 — Supplemental Figures [file 41416_2021_1270_MOESM1_ESM.pptx]

## Slide 1
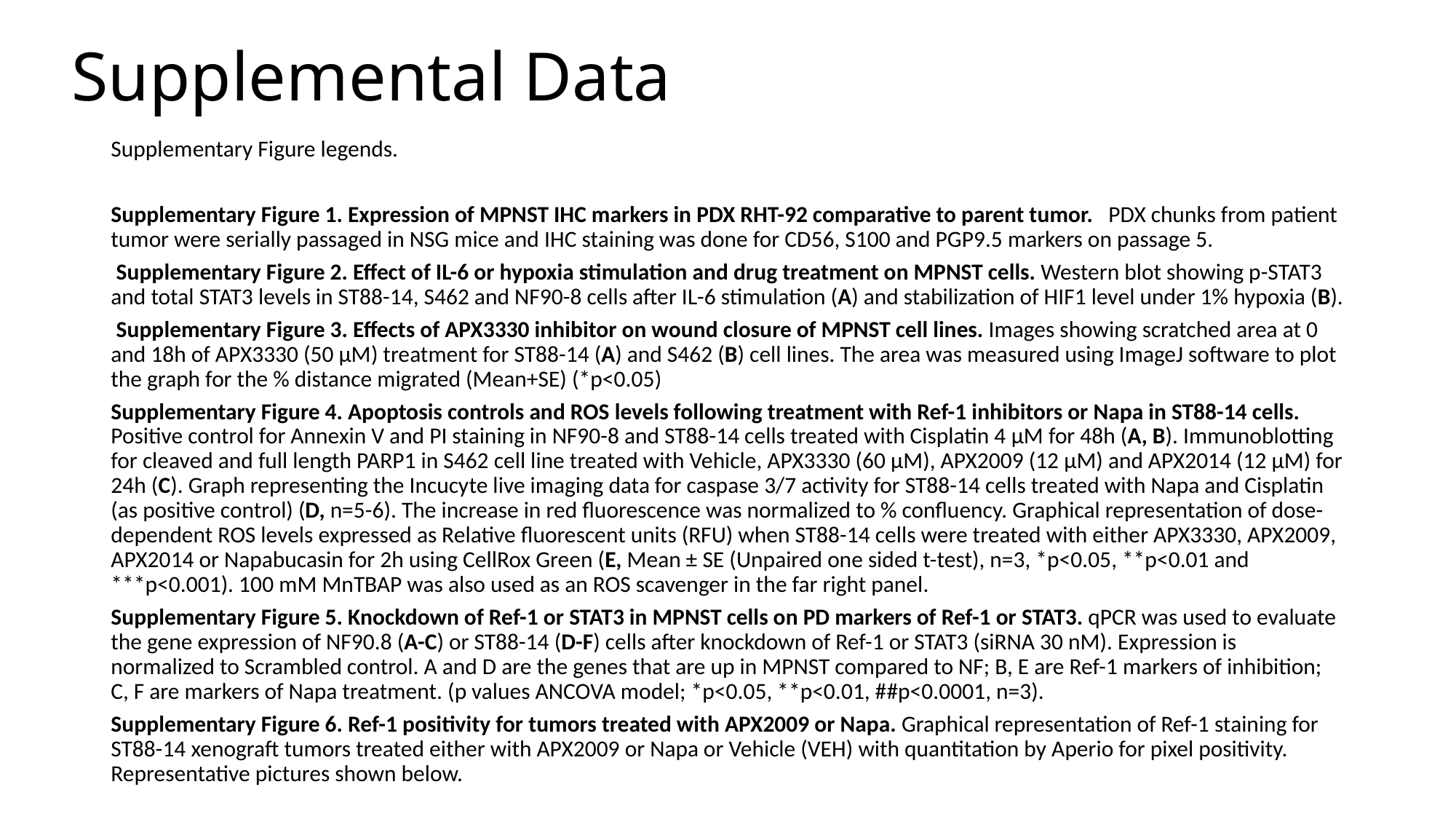

# Supplemental Data
Supplementary Figure legends.
Supplementary Figure 1. Expression of MPNST IHC markers in PDX RHT-92 comparative to parent tumor. PDX chunks from patient tumor were serially passaged in NSG mice and IHC staining was done for CD56, S100 and PGP9.5 markers on passage 5.
 Supplementary Figure 2. Effect of IL-6 or hypoxia stimulation and drug treatment on MPNST cells. Western blot showing p-STAT3 and total STAT3 levels in ST88-14, S462 and NF90-8 cells after IL-6 stimulation (A) and stabilization of HIF1 level under 1% hypoxia (B).
 Supplementary Figure 3. Effects of APX3330 inhibitor on wound closure of MPNST cell lines. Images showing scratched area at 0 and 18h of APX3330 (50 µM) treatment for ST88-14 (A) and S462 (B) cell lines. The area was measured using ImageJ software to plot the graph for the % distance migrated (Mean+SE) (*p<0.05)
Supplementary Figure 4. Apoptosis controls and ROS levels following treatment with Ref-1 inhibitors or Napa in ST88-14 cells. Positive control for Annexin V and PI staining in NF90-8 and ST88-14 cells treated with Cisplatin 4 µM for 48h (A, B). Immunoblotting for cleaved and full length PARP1 in S462 cell line treated with Vehicle, APX3330 (60 µM), APX2009 (12 µM) and APX2014 (12 µM) for 24h (C). Graph representing the Incucyte live imaging data for caspase 3/7 activity for ST88-14 cells treated with Napa and Cisplatin (as positive control) (D, n=5-6). The increase in red fluorescence was normalized to % confluency. Graphical representation of dose-dependent ROS levels expressed as Relative fluorescent units (RFU) when ST88-14 cells were treated with either APX3330, APX2009, APX2014 or Napabucasin for 2h using CellRox Green (E, Mean ± SE (Unpaired one sided t-test), n=3, *p<0.05, **p<0.01 and ***p<0.001). 100 mM MnTBAP was also used as an ROS scavenger in the far right panel.
Supplementary Figure 5. Knockdown of Ref-1 or STAT3 in MPNST cells on PD markers of Ref-1 or STAT3. qPCR was used to evaluate the gene expression of NF90.8 (A-C) or ST88-14 (D-F) cells after knockdown of Ref-1 or STAT3 (siRNA 30 nM). Expression is normalized to Scrambled control. A and D are the genes that are up in MPNST compared to NF; B, E are Ref-1 markers of inhibition; C, F are markers of Napa treatment. (p values ANCOVA model; *p<0.05, **p<0.01, ##p<0.0001, n=3).
Supplementary Figure 6. Ref-1 positivity for tumors treated with APX2009 or Napa. Graphical representation of Ref-1 staining for ST88-14 xenograft tumors treated either with APX2009 or Napa or Vehicle (VEH) with quantitation by Aperio for pixel positivity. Representative pictures shown below.

## Slide 2
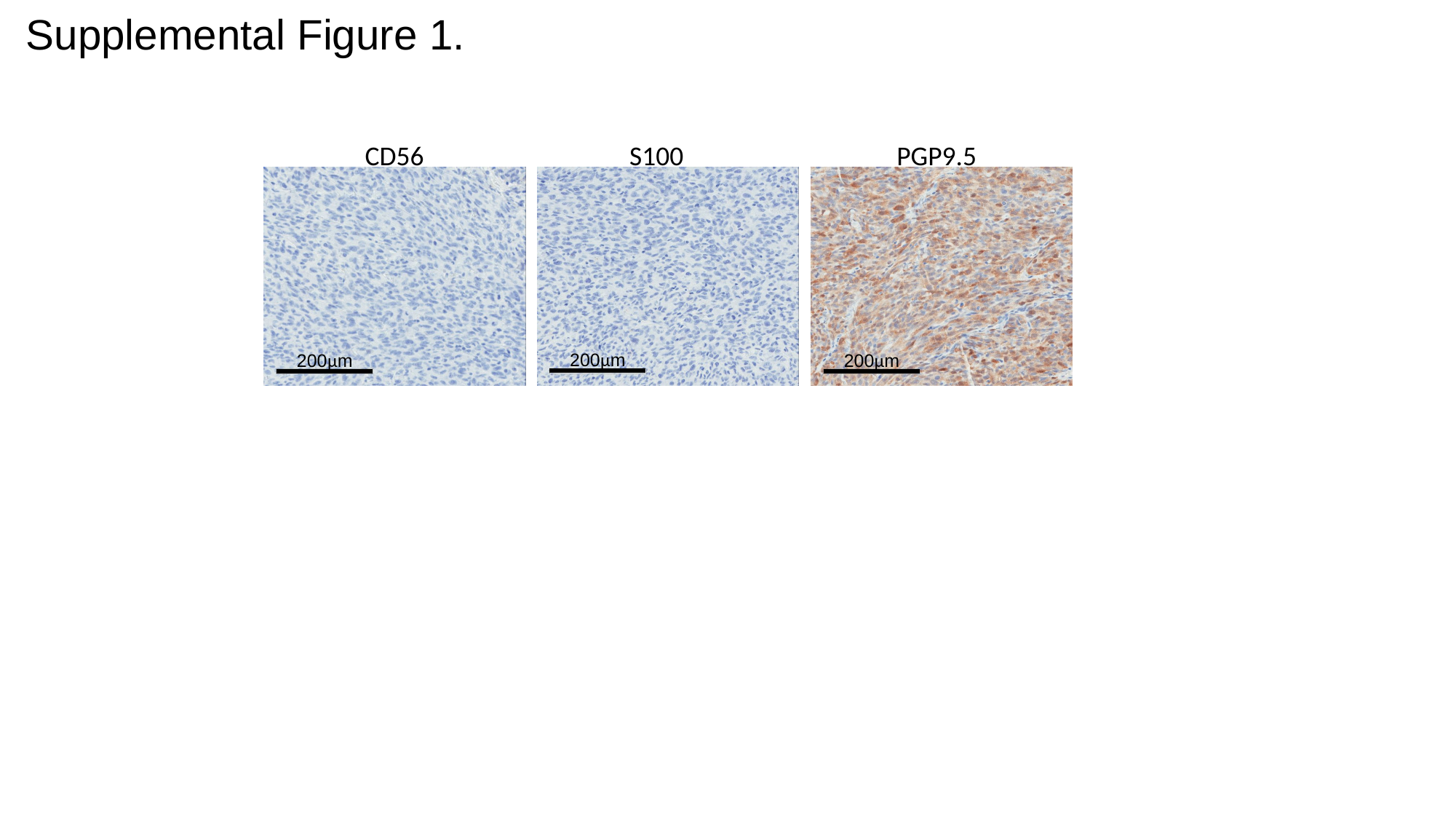

# Supplemental Figure 1.
CD56
S100
PGP9.5
200µm
200µm
200µm

## Slide 3
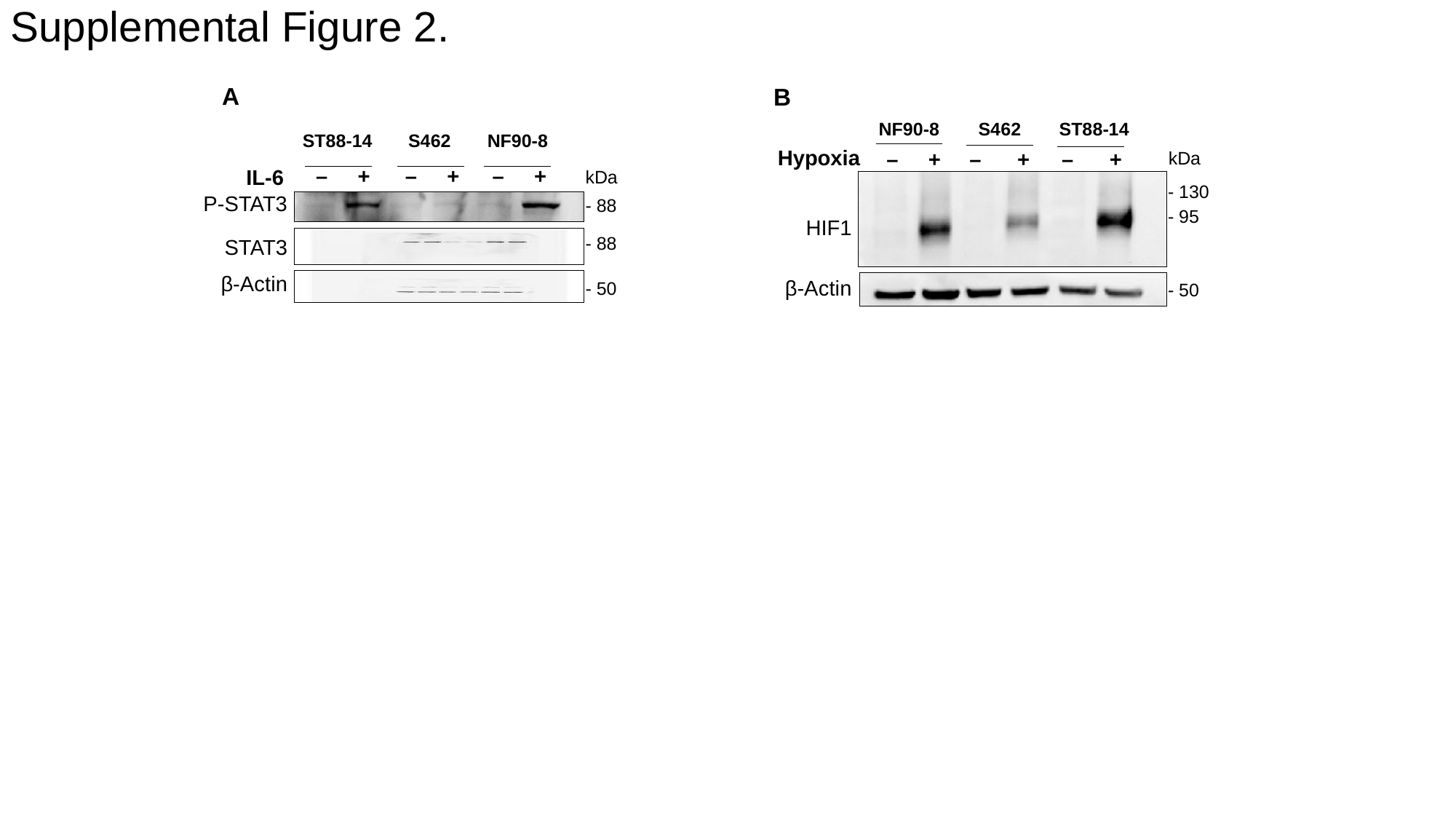

Supplemental Figure 2.
A
B
NF90-8
S462
ST88-14
Hypoxia
– +
– +
– +
kDa
- 130
- 95
HIF1
β-Actin
- 50
ST88-14
S462
NF90-8
– +
– +
– +
IL-6
kDa
P-STAT3
- 88
- 88
STAT3
β-Actin
- 50

## Slide 4
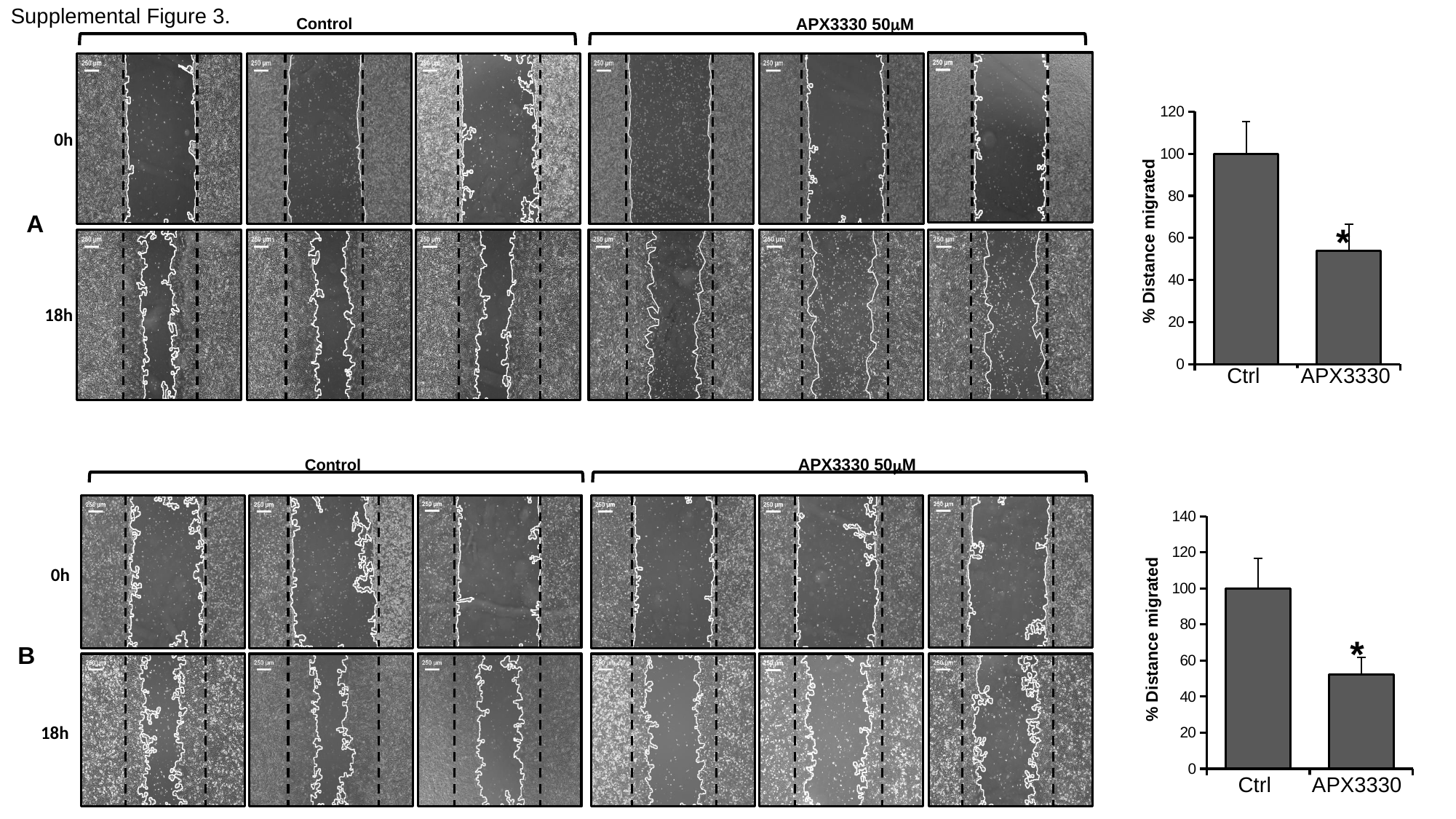

Supplemental Figure 3.
APX3330 50mM
Control
0h
18h
### Chart
| Category | |
|---|---|
| UT | 100.0 |
| E3330 | 53.7795145148961 |*
% Distance migrated
59%
Ctrl APX3330
A
APX3330 50mM
Control
0h
18h
### Chart
| Category | |
|---|---|
| UT | 100.0 |
| E3330 | 52.44323584956698 |% Distance migrated
*
Ctrl APX3330
B

## Slide 5
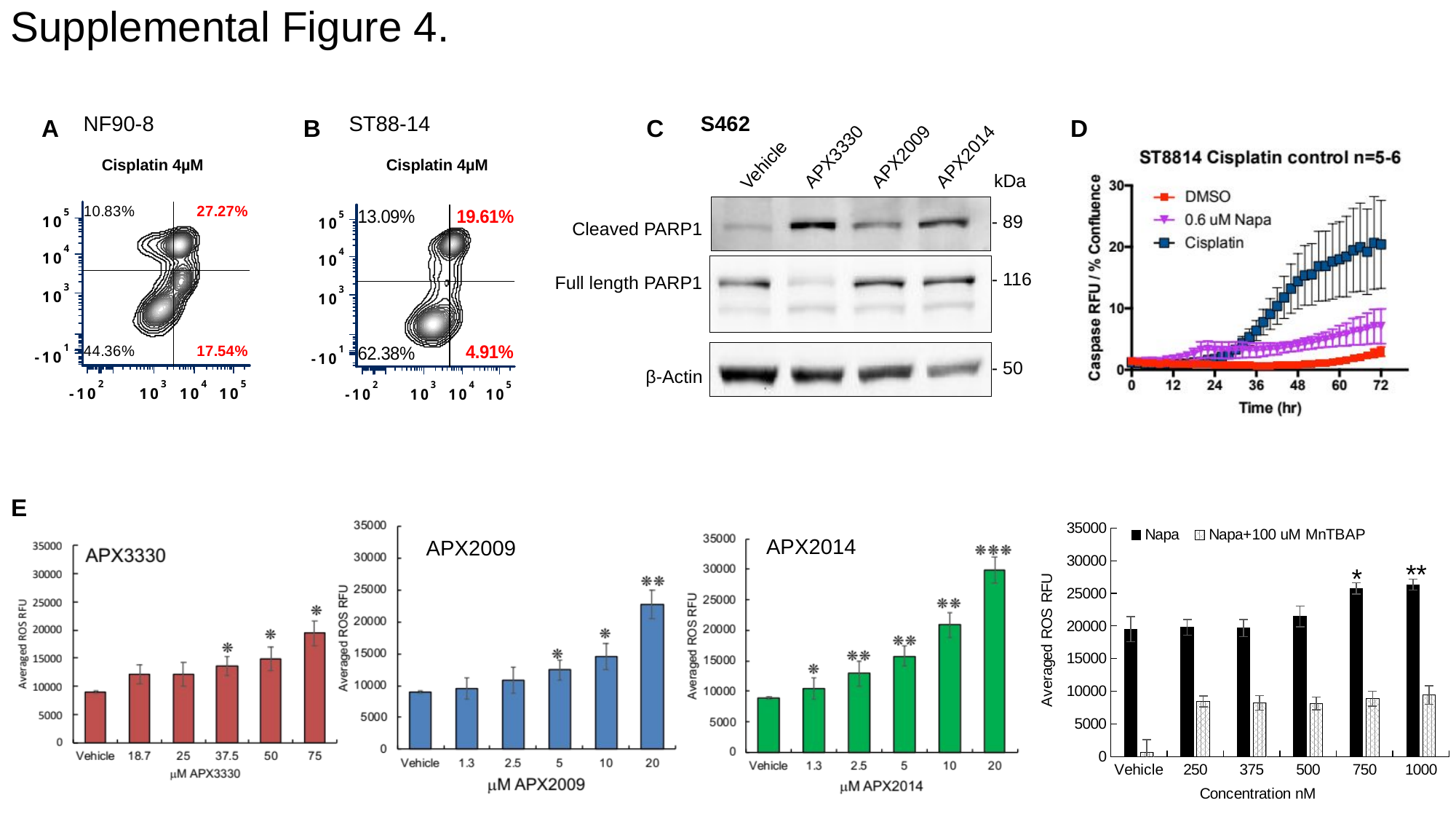

Supplemental Figure 4.
APX3330
APX2009
APX2014
Vehicle
kDa
- 89
Cleaved PARP1
- 116
Full length PARP1
- 50
β-Actin
NF90-8
ST88-14
S462
A
B
C
D
Cisplatin 4µM
Cisplatin 4µM
E
### Chart
| Category | Napa | Napa+100 uM MnTBAP |
|---|---|---|
| Vehicle | 19540.125 | 691.7499999999994 |
| 250 | 19801.41666666666 | 8469.354166666662 |
| 375 | 19679.16666666666 | 8260.02083333333 |
| 500 | 21470.83333333334 | 8184.354166666668 |
| 750 | 25768.91666666666 | 8895.604166666666 |
| 1000 | 26321.25 | 9430.020833333328 |**
*
APX2014
APX2009

## Slide 6
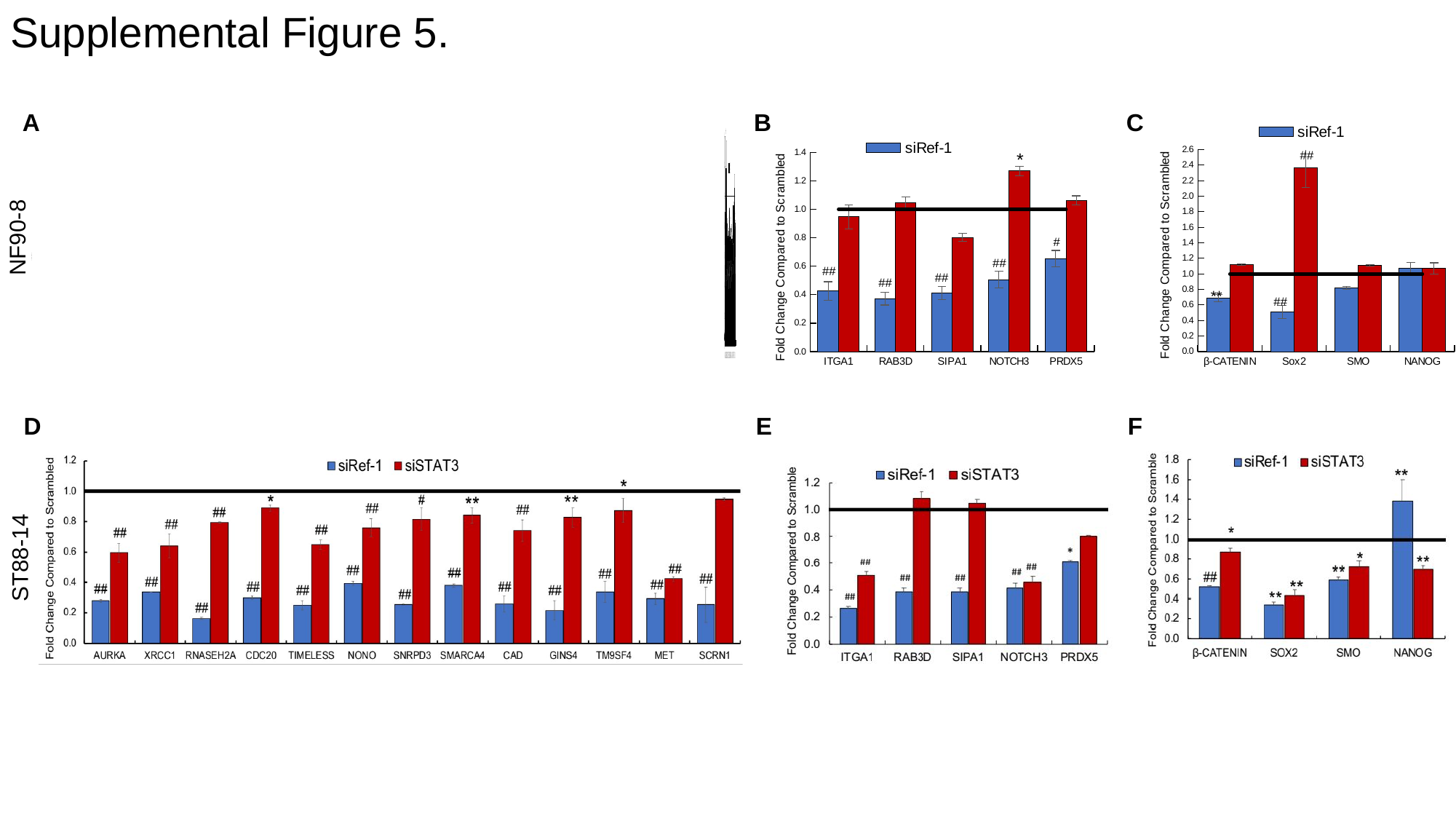

Supplemental Figure 5.
A
### Chart
| Category | siRef-1 | siSTAT3 | SCR |
|---|---|---|---|
| AURKA | 0.6078162843838911 | 0.8941632199402757 | 1.0 |
| XRCC1 | 0.4383226941045668 | 1.1816550736799718 | 1.0 |
| RNASEH2A | 0.47504526818355025 | 0.8153456398906647 | 1.0 |
| CDC20 | 0.5179618142110831 | 0.7297562634695215 | 1.0 |
| TIMELESS | 0.4334149885017258 | 0.7666395665218751 | 1.0 |
| NONO | 0.5157639638865463 | 0.9165325369546179 | 1.0 |
| SNRPD3 | 0.4225285434238968 | 0.7249236723883602 | 1.0 |
| SMARCA4 | 0.5379623412793038 | 0.7874702631685908 | 1.0 |
| CAD | 0.48159195110903247 | 0.7131823104820377 | 1.0 |
| GINS4 | 0.5152829661734639 | 0.6808822268585842 | 1.0 |
| TM9SF4 | 0.6301484116691104 | 0.9371933706985546 | 1.0 |
| MET | 0.8004507255930081 | 1.0529986769468223 | 1.0 |
| SCRN1 | 0.5393972005834126 | 0.8217897871244043 | 1.0 |B
### Chart
| Category | siRef-1 | siSTAT3 | SCR |
|---|---|---|---|
| ITGA1 | 0.42612571842934605 | 0.9490472096316823 | 1.0 |
| RAB3D | 0.3731220982752596 | 1.0458506868040913 | 1.0 |
| SIPA1 | 0.41428535871526434 | 0.8038225101159601 | 1.0 |
| NOTCH3 | 0.5070607525278933 | 1.2709912014960645 | 1.0 |
| PRDX5 | 0.6544277327227523 | 1.065339005684564 | 1.0 |C
### Chart
| Category | siRef-1 | siSTAT3 | SCR |
|---|---|---|---|
| β-CATENIN | 0.6897006240003029 | 1.119235509114491 | 1.0 |
| Sox2 | 0.5139407052216666 | 2.364693733379123 | 1.0 |
| SMO | 0.8220766005097996 | 1.1089406537241397 | 1.0 |
| NANOG | 1.068528321024643 | 1.068364011409343 | 1.0 |NF90-8
D
E
F
ST88-14

## Slide 7
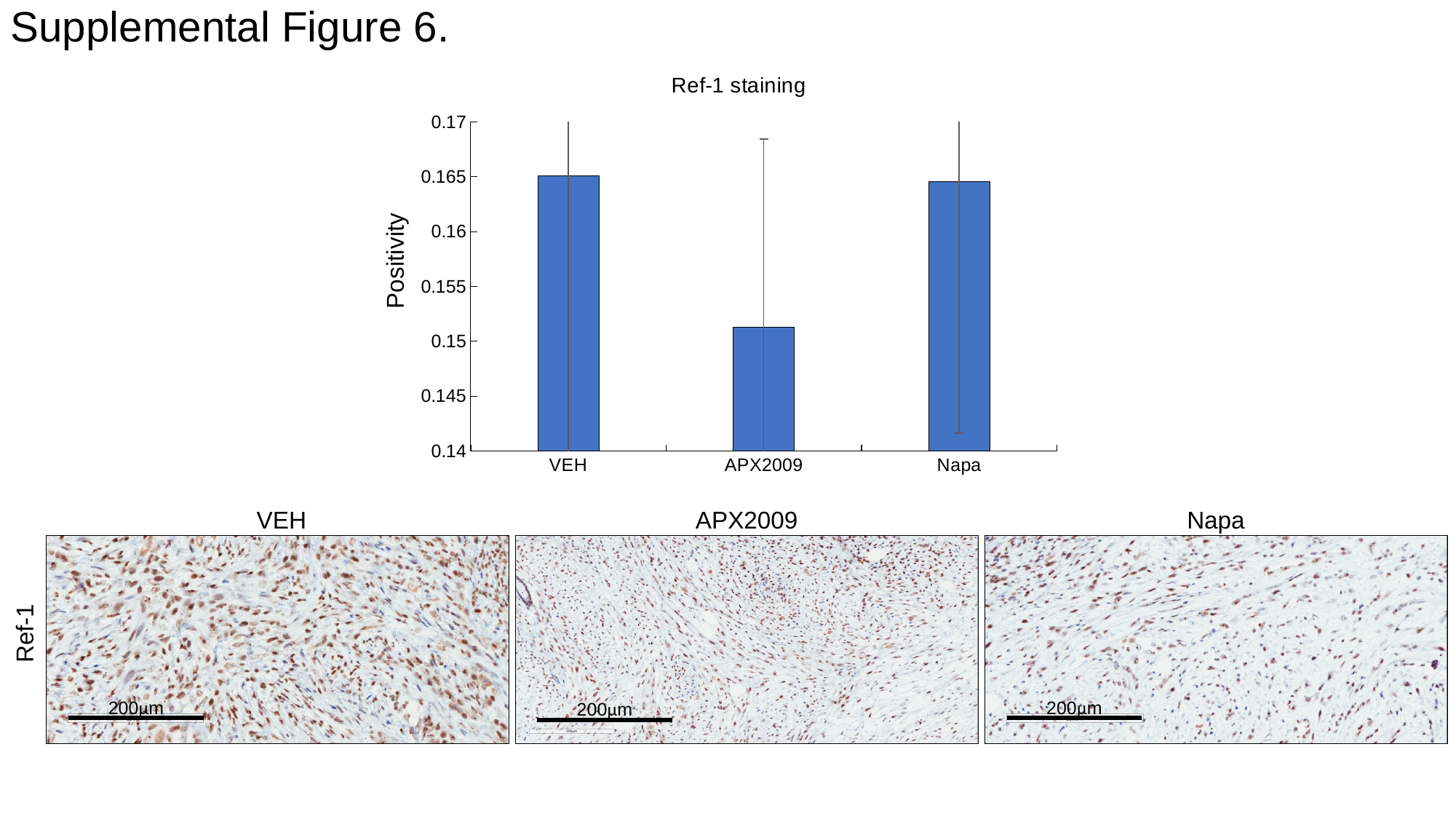

Supplemental Figure 6.
### Chart: Ref-1 staining
| Category | |
|---|---|
| VEH | 0.1650925 |
| APX2009 | 0.1512869 |
| Napa | 0.1645388125 |Positivity
VEH
APX2009
Napa
Ref-1
200µm
200µm
200µm
